# Supplementary material for: Efficacy of Kan Jang® in Patients with Mild COVID-19: A Randomized, Quadruple-Blind, Placebo-Controlled Trial
Source: Pharmaceuticals (Basel). 2023 Aug 22;16(9):1196. doi: 10.3390/ph16091196 (PMC10535596; doi:10.3390/ph16091196)
Supplement: Supplementary file 1 [file pharmaceuticals-16-01196-s001.zip › Supplement 2 Figures S1-S57.pdf]

The rate of cases turning to severe

- The Rate of Patients with Clinical Deterioration and Duration of Hospitalization in the Treatment Group and Control Group
- In Kan Jang®, Group A of 68 patients, 12 were dropouts, and three were withdrawn from the study due to lack of efficacy and disease progression; they continued the treatment with steroids and antibiotics. Figure 1.
- In Placebo Group B, of 72 patients, 16 were dropouts, and ten were withdrawn from the study due to lack of efficacy and disease progression; they continued the treatment with steroids and antibiotics. Figure 1.
- Therefore:
    - Group A's (Kan Jang, 56 patients) disease progression rate (5.36%).
    - Group B's (placebo, 56 patients) disease progression rate (17.86%).
- Where p = 0.0176 (significant result) at 95% significance level (Confidence 95%), power 90.15%.
- Absolute risk reduction by Kan Jang treatment is 12.5% (ARR=0.125),
- Relative risk/odds ratio = 0.3
- Relative risk reduction (RRR,%) = 70 %.
- The number of patients required to treat with Kan Jang to prevent one additional bad outcome (defined as the Number Needed to Treat, NNT = 1/ARR = 8) comprises 8.
- The higher ARR and lower NNT are, the more effective the intervention is.
- Relative risk, relative and absolute risk reduction, number needed to treat and confidence intervals - Smart Health Choices - NCBI Bookshelf (nih.gov)

The rate of cases turning to severe

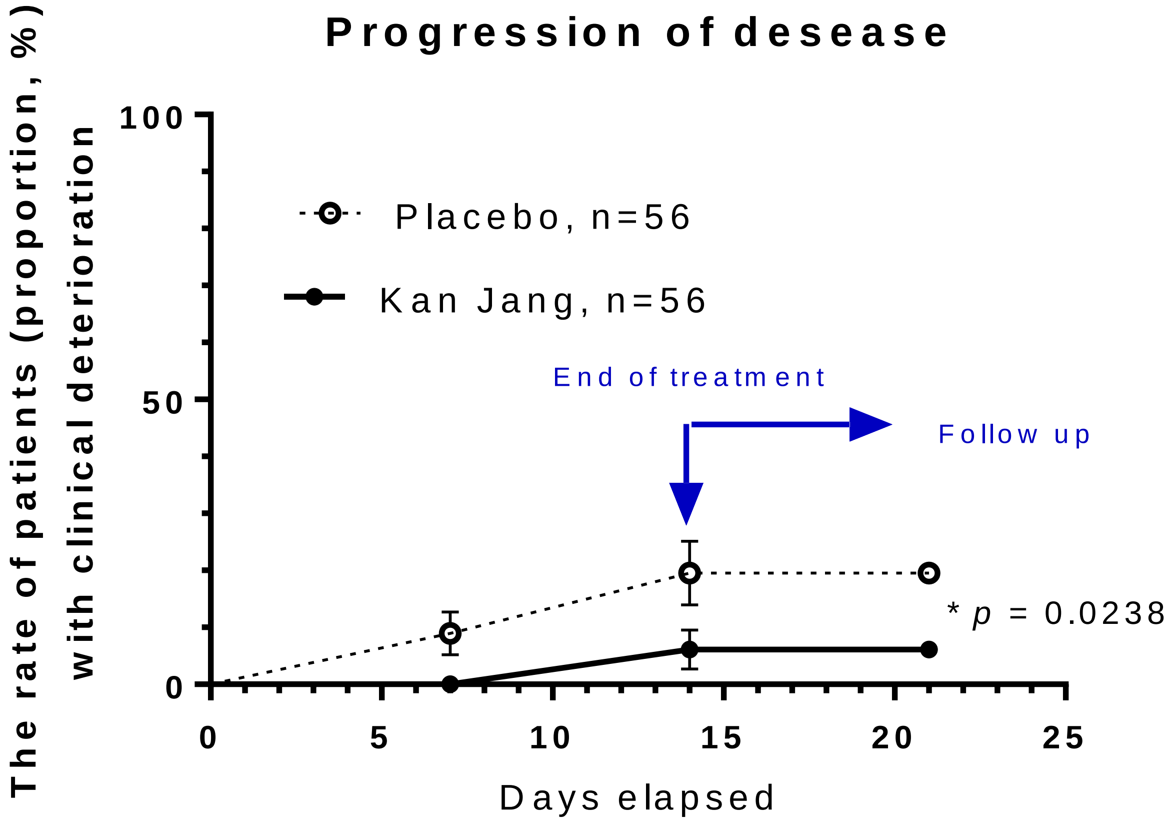

Comparison of Survival Curves		
Log-rank (Mantel-Cox) test		
Chi square	4,648	
df	1	
P value	0,0311	
P value summary	*	
Are the survival curves sig different?	Yes	
Gehan-Breslow-Wilcoxon test		
Chi square	5,112	
df	1	
P value	0,0238	
P value summary	*	
Are the survival curves sig different?	Yes	
Median survival		
Kan Jang, n=34	Undefined	
Placebo, n=52	Undefined	
Hazard Ratio (Mantel-Haenszel)	A/B	B/A
Ratio (and its reciprocal)	0,2906	3,441
95% CI of ratio	0,09448 to 0,8938	1,119 to 10,58
Hazard Ratio (logrank)	A/B	B/A
Ratio (and its reciprocal)	0,2768	3,612
95% CI of ratio	0,09789 to 0,8627	1,159 to 10,22

The rate of cases turning to severe, PPA

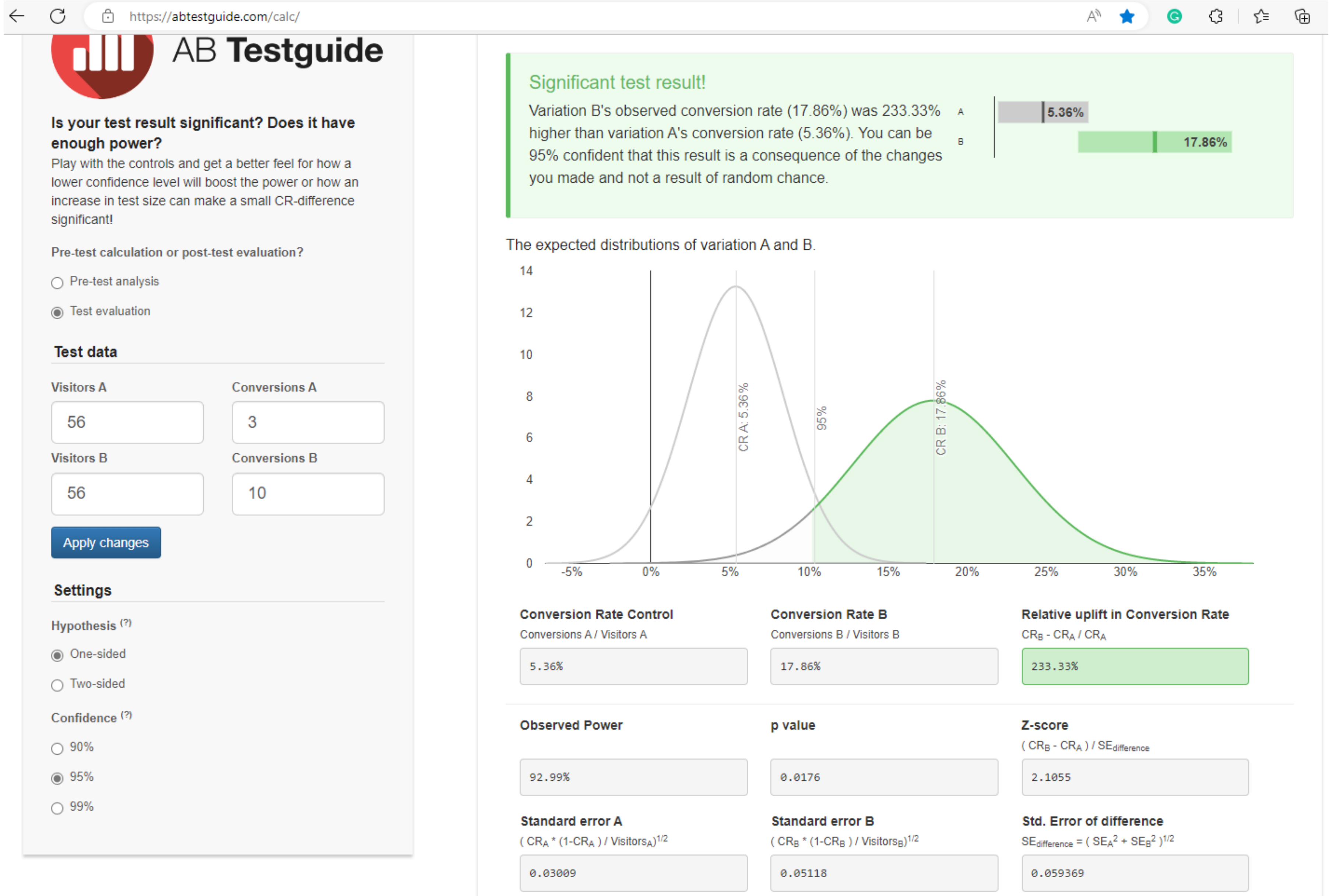

The rate of cases turning to severe, ITT analysis

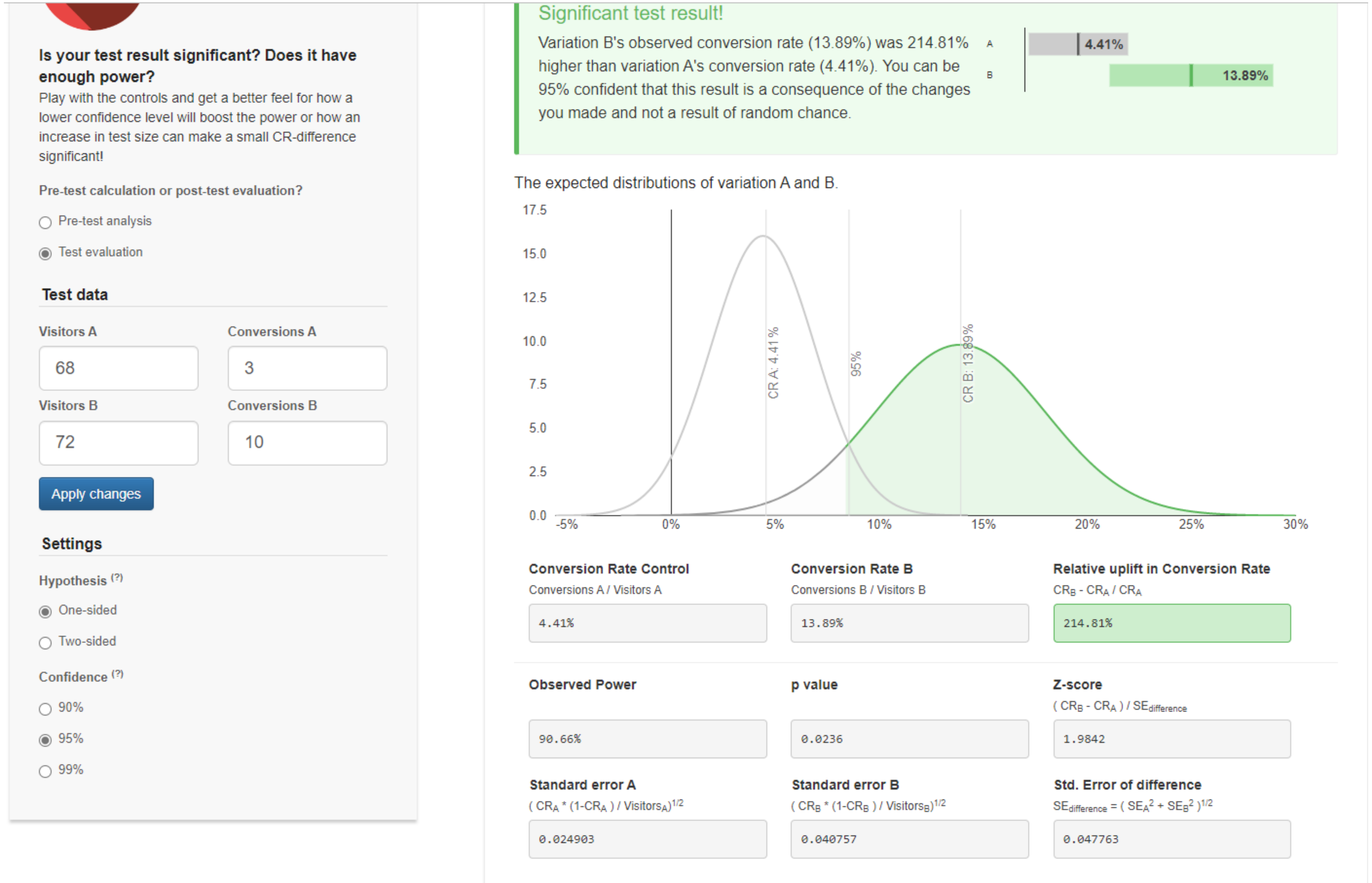

The overall clinical effectiveness defined as the ratio of effective to ineffective cases vs placebo control

- Group A (Kan Jang, 56 patients) disease progression rate (5.36%).
- Group B's (placebo, 56 patients) disease progression rate (17.86%).
- Group A: Absolute effectiveness - 94,56%
  - Within group ratio of effective to ineffective cases 17.65
- Group B: Absolute effectiveness - 82,14%
- Relative effectiveness 4.6
  - Within group ratio of effective to ineffective cases 4.6
- Overall clinical effectiveness *vs* placebo control = 3.84
- A/B-Test Calculator - Power & Significance - ABTestGuide.com

Days at clinic or home

ITT analysis in 140 patients

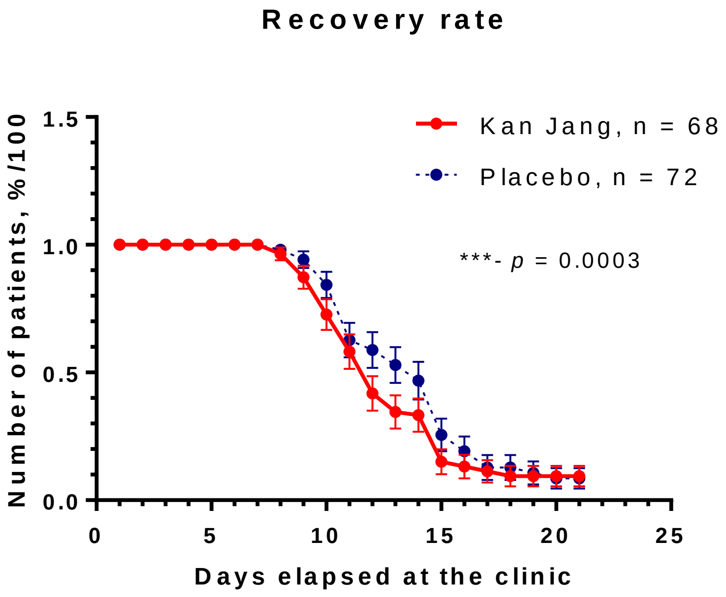

PP analysis in 100 patients

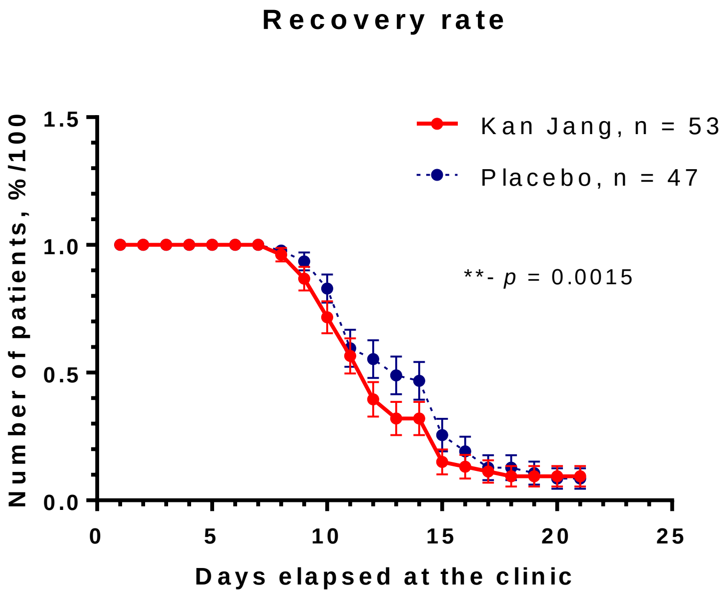

Days at clinic or home

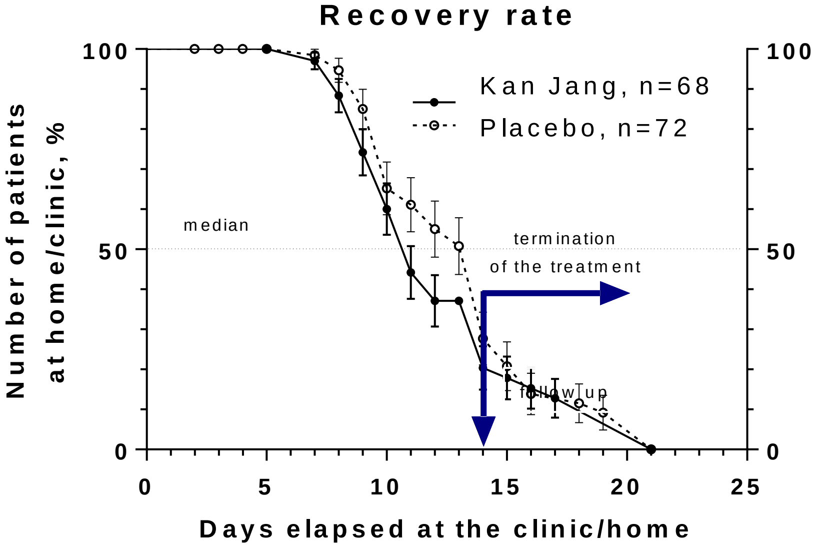

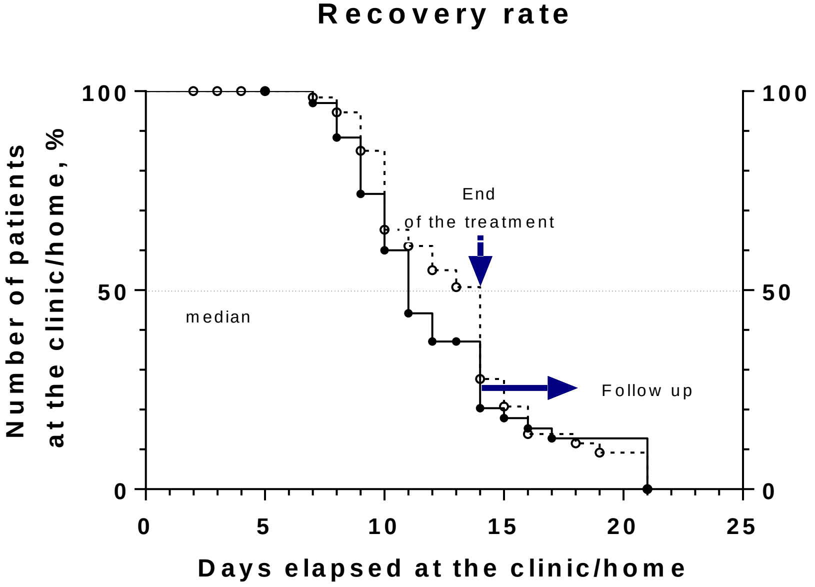

Days elapsed	Kan Jang, n=68		Placebo, n=72	
	%	SEM	%	SEM
0	100		100	
2			100	
3			100	
4			100	
5	100		100	
7	97,0	2,1	98,5	1,5
8	88,4	4,2	94,7	3,0
9	74,2	5,8	85,0	4,9
10	60,1	6,5	65,2	6,6
11	44,2	6,6	61,2	6,8
12	37,1	6,4	55,0	7,0
13	37,1		50,8	7,1
14	20,4	5,4	27,7	6,6
15	17,9	5,3	20,8	6,1
16	15,3	5,1	13,9	5,2
17	12,8	4,9		
18			11,5	4,8
19			9,2	4,4
21	0	0	0	0

Median at clinic /home		
Kan Jang, n=68	11	
Placebo, n=72	14	
Ratio (and its reciprocal)	0,7857	1,273
95% CI of ratio	0,5305 to 1,164	0,8594 to 1,885
Hazard Ratio (Mantel-Haenszel)	A/B	B/A
Ratio (and its reciprocal)	1,232	0,8117
95% CI of ratio	0,7780 to 1,951	0,5126 to 1,285

Negative conversion rate of SARS CoV-2 virus detection

ITT analysis in 140 patients

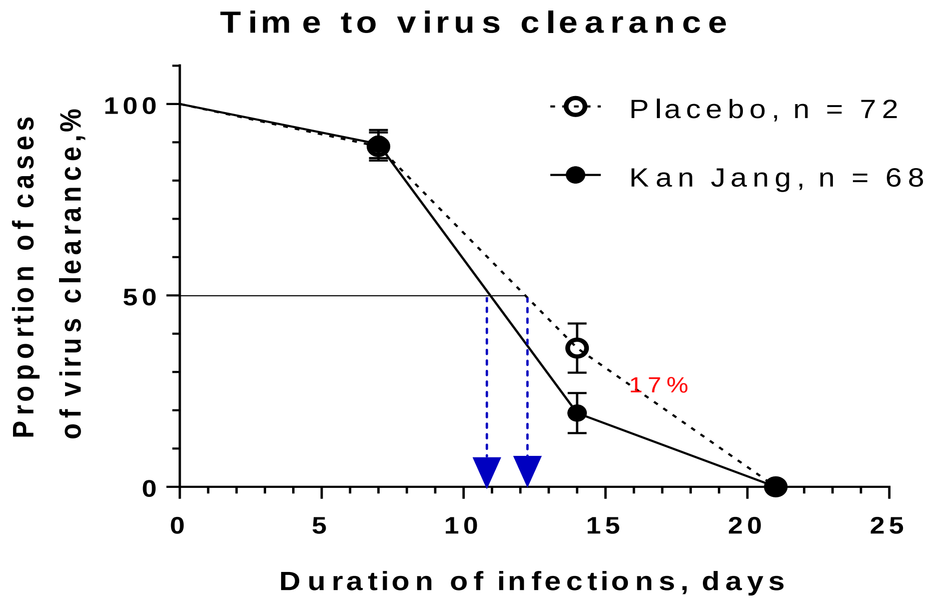

Hazard Ratio, Kan Jang/Placebo - 1,69
95% CI of ratio: from 0,8698 to 3,269

Days elapsed	Kan Jang, n=68		Placebo, n=72	
0	100	SEM	100	SEM
7	89,6	3,7	88,9	3,7
14	19,3	5,2	36,3	6,4
21	0	0	0	0

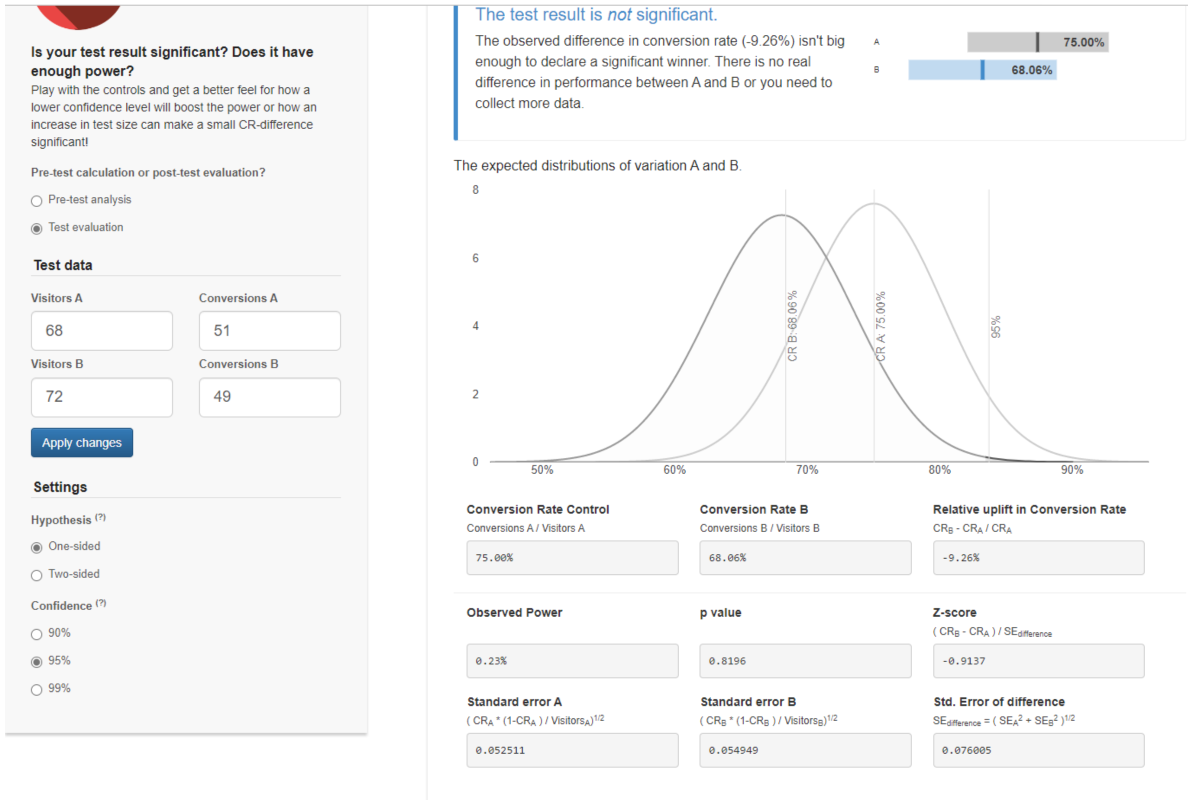

Disappearance rate of symptoms Cough

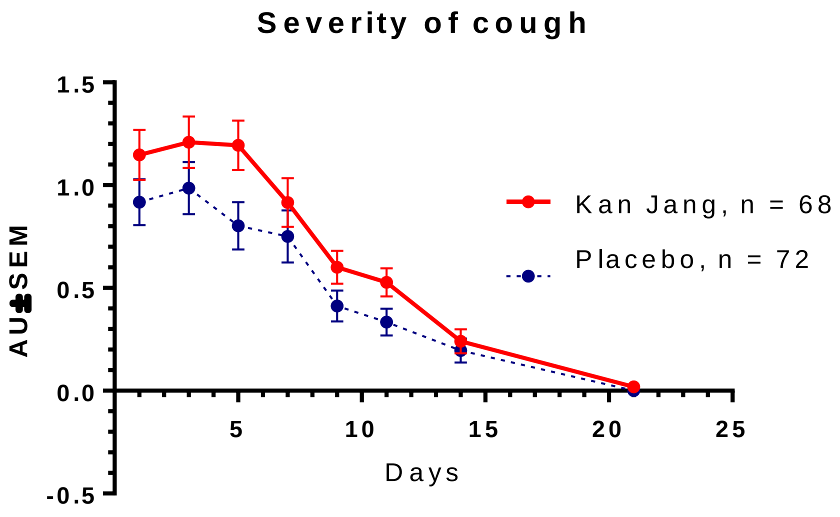

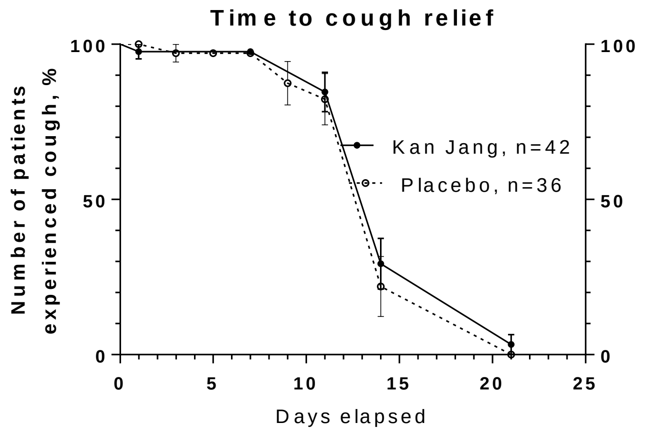

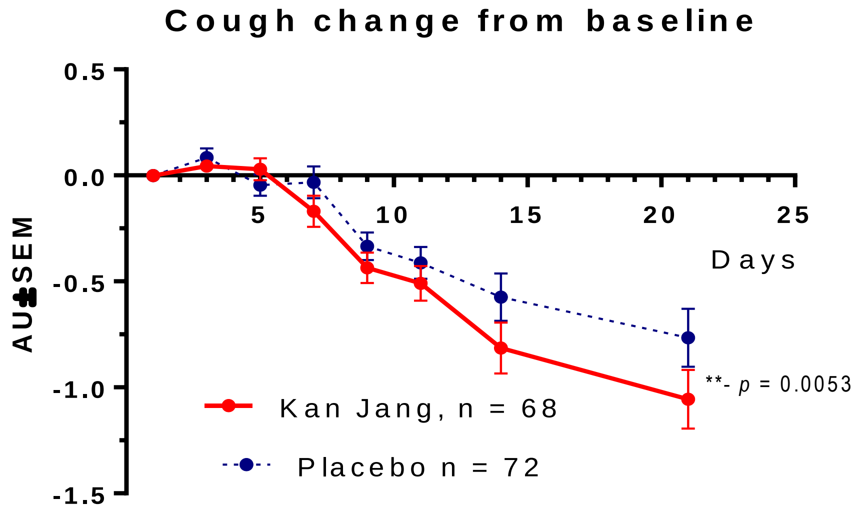

ITT analysis in 140 patients

Days	Kan Jang, n = 68			Placebo, n=72		
	Mean	SD	n	Mean	SD	n
1	0	0	68	0	0	72
3	0,045	0,025	67	0,085	0,044	71
5	0,030	0,052	67	-0,045	0,050	66
7	-0,169	0,073	59	-0,033	0,075	60
9	-0,436	0,072	55	-0,333	0,067	51
11	-0,509	0,082	55	-0,412	0,075	51
14	-0,815	0,121	54	-0,574	0,113	47
21	-1,057	0,138	53	-0,766	0,137	47

Cough

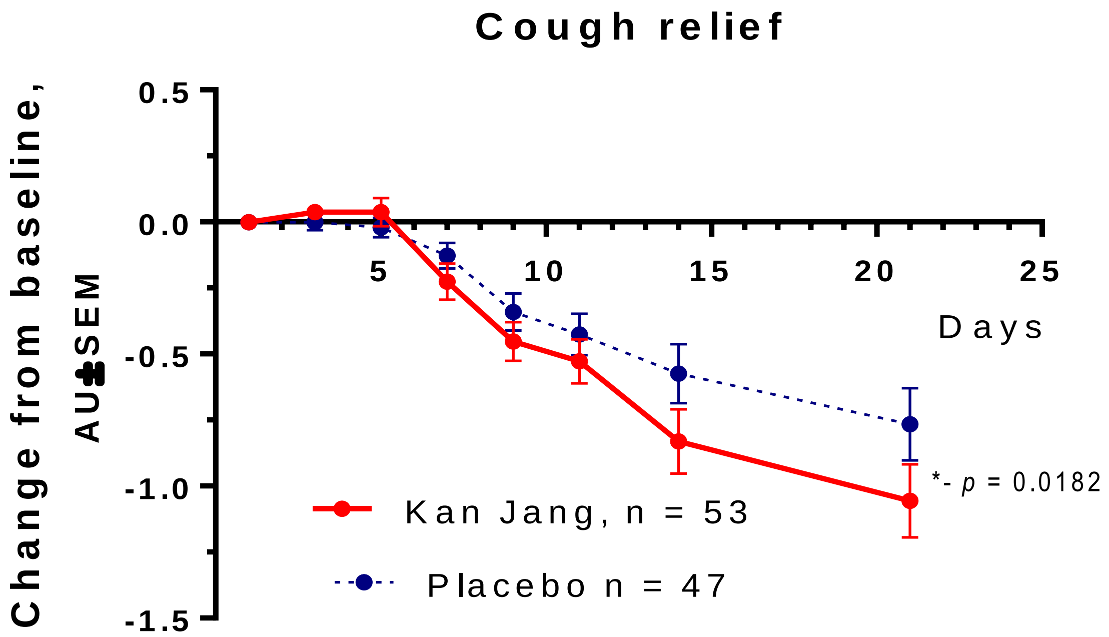

PP analysis in 100 patients

Days	Kan Jang, n = 53			Placebo, n=47		
	Mean	SD	n	Mean	SD	n
1	0	0	53	0	0	47
3	0,038	0,026	53	0,000	0,030	47
5	0,038	0,054	53	-0,021	0,037	47
7	-0,226	0,069	53	-0,128	0,049	47
9	-0,453	0,074	53	-0,340	0,070	47
11	-0,528	0,083	53	-0,426	0,079	47
14	-0,830	0,123	53	-0,574	0,113	47
21	-1,057	0,138	53	-0,766	0,137	47

Cough

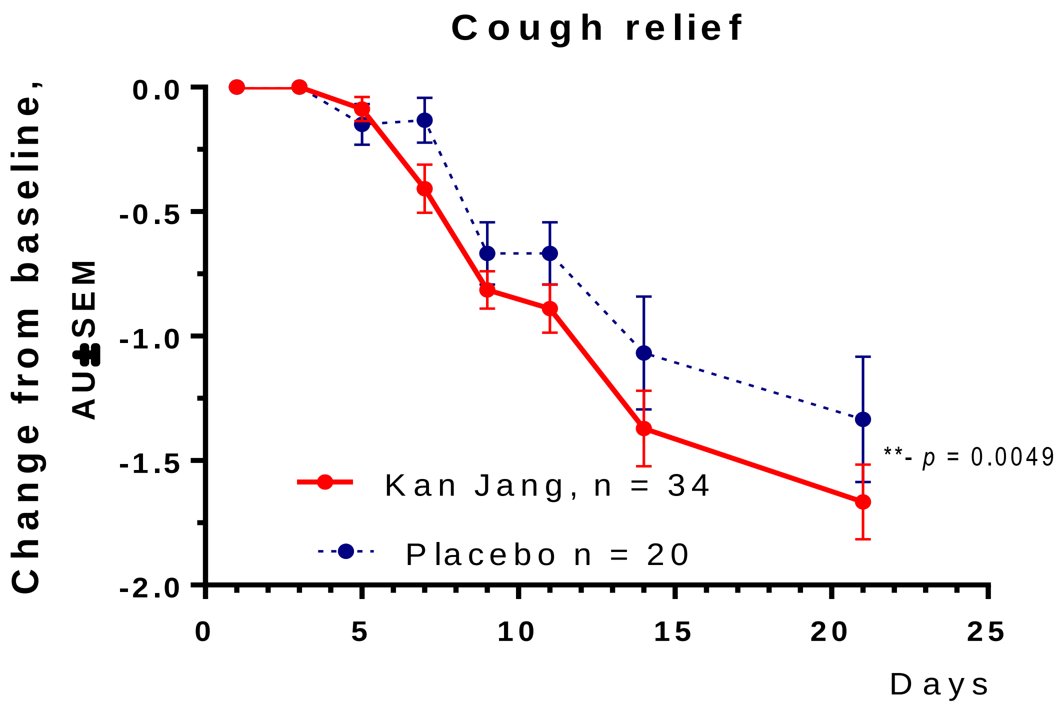

ITT analysis in a subgroup of 54 patients

Days	Kan Jang, n = 34			Placebo, n=20		
	Mean	SD	n	Mean	SD	n
1	0	0	34	0	0	20
3	0	0	34	0	0	20
5	-0,088	0,049	34	-0,150	0,082	20
7	-0,407	0,096	27	-0,133	0,091	15
9	-0,815	0,076	27	-0,667	0,126	15
11	-0,889	0,097	27	-0,667	0,126	15
14	-1,370	0,152	27	-1,067	0,228	15
21	-1,667	0,151	27	-1,333	0,252	15

Cough

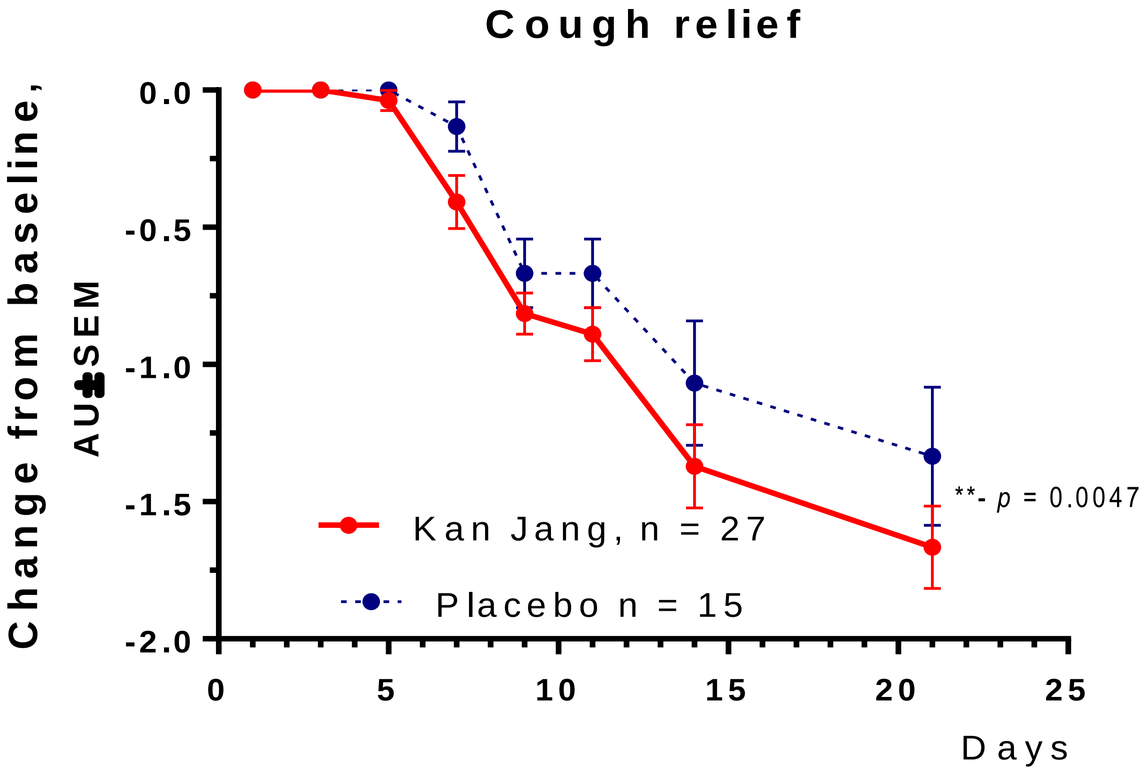

PP analysis in a subgroup of 42 patients

Days	Kan Jang, n = 27			Placebo, n=15		
	Mean	SD	n	Mean	SD	n
1	0	0	27	0	0	15
3	0	0	27	0	0	15
5	-0,037	0,037	27	0	0	15
7	-0,407	0,096	27	-0,133	0,091	15
9	-0,815	0,076	27	-0,667	0,126	15
11	-0,889	0,097	27	-0,667	0,126	15
14	-1,370	0,152	27	-1,067	0,228	15
21	-1,667	0,151	27	-1,333	0,252	15

Sore throat pain

ITT analysis in 140 patients

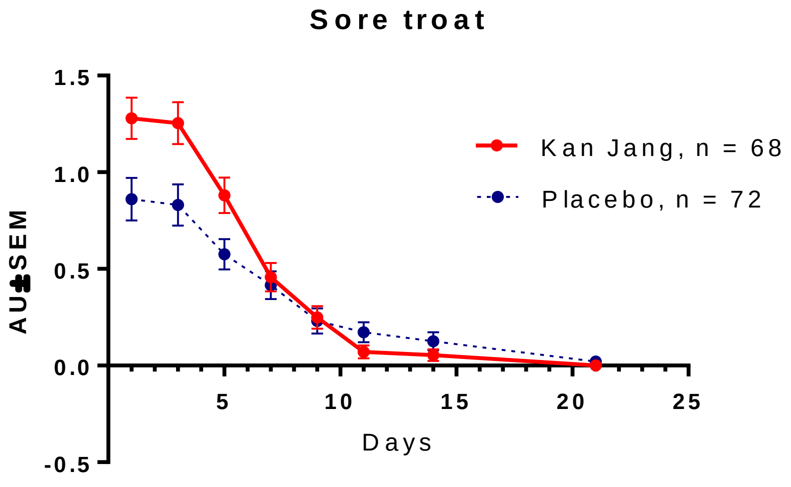

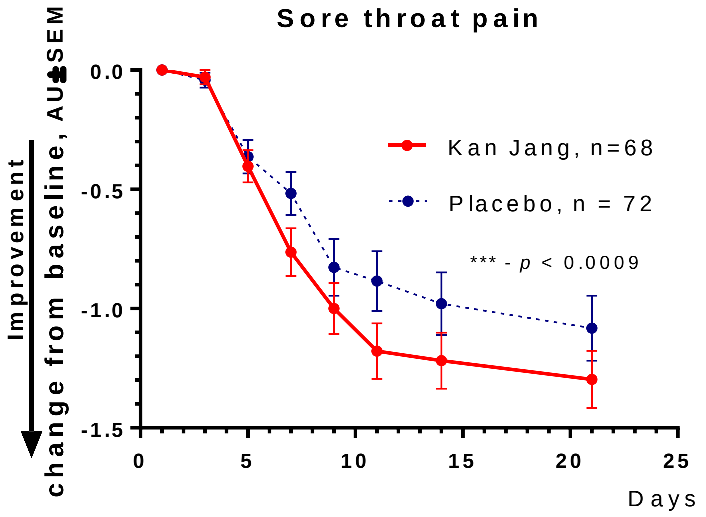

Days	Kan Jang, n = 68			Placebo, n=72		
	Mean	SD	n	Mean	SD	n
1	0	0	68	0	0	72
3	-0,030	0,030	67	-0,04	0,03	71
5	-0,403	0,067	67	-0,36	0,07	66
7	-0,763	0,101	59	-0,52	0,09	60
9	-1,000	0,108	56	-0,83	0,12	52
11	-1,179	0,117	56	-0,88	0,12	52
14	-1,218	0,118	55	-0,98	0,13	49
21	-1,296	0,120	54	-1,08	0,14	49

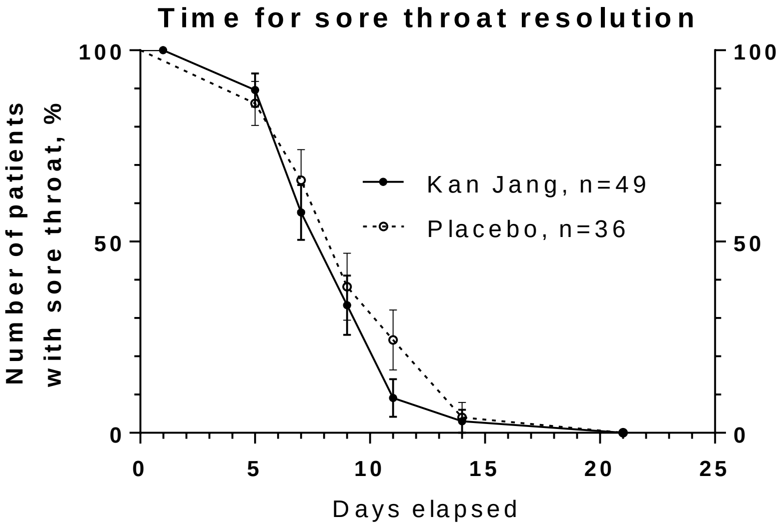

Days elapsed	Kan Jang, n=68		Placebo, n=72	
0	100		100	
1	100			
5	89,583	4,409	86,111	5,764
7	57,589	7,204	66,019	7,984
9	33,341	7,743	38,221	8,791
11	9,093	4,950	24,323	7,876
14	3,031	2,974	4,054	3,926
21	0	0	0	0

Sore throat pain

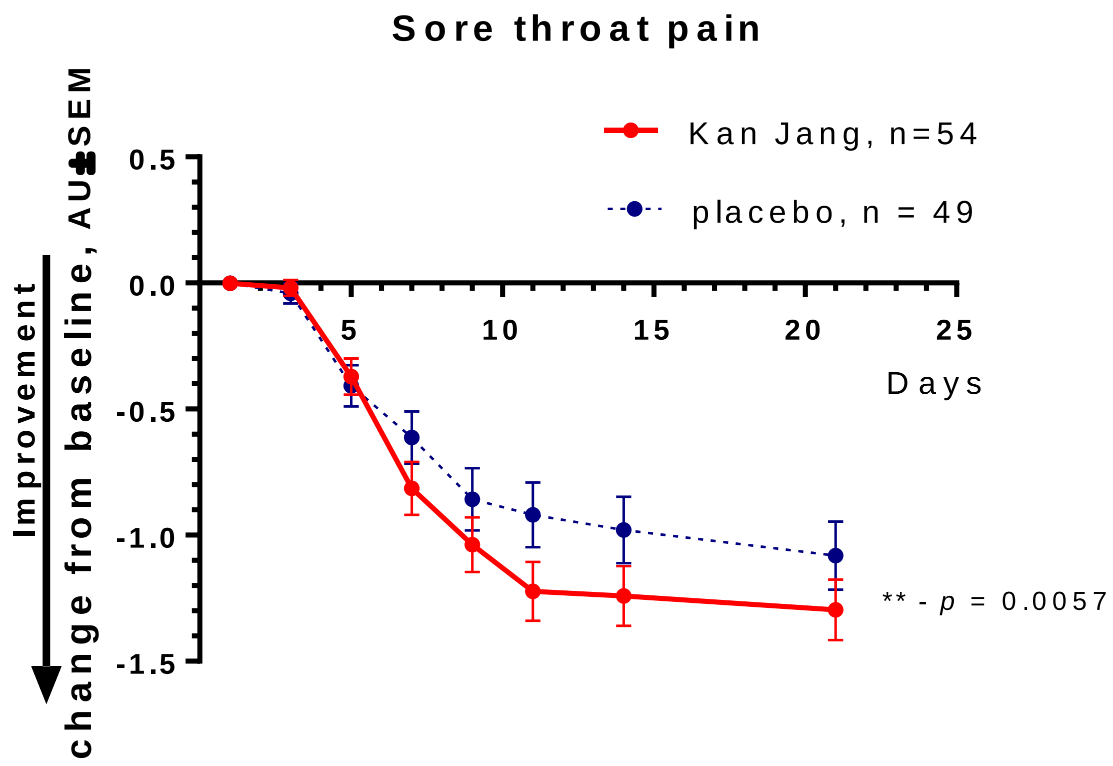

PP analysis in 103 patients

Days	Kan Jang, n = 54			Placebo, n=49		
	Mean	SD	n	Mean	SD	n
1	0	0	54	0	0	49
3	-0,019	0,032	54	-0,04	0,04	49
5	-0,370	0,071	54	-0,41	0,08	49
7	-0,815	0,106	54	-0,61	0,10	49
9	-1,037	0,109	54	-0,86	0,12	49
11	-1,222	0,117	54	-0,92	0,13	49
14	-1,241	0,118	54	-0,98	0,13	49
21	-1,296	0,120	54	-1,08	0,14	49

Sore throat pain

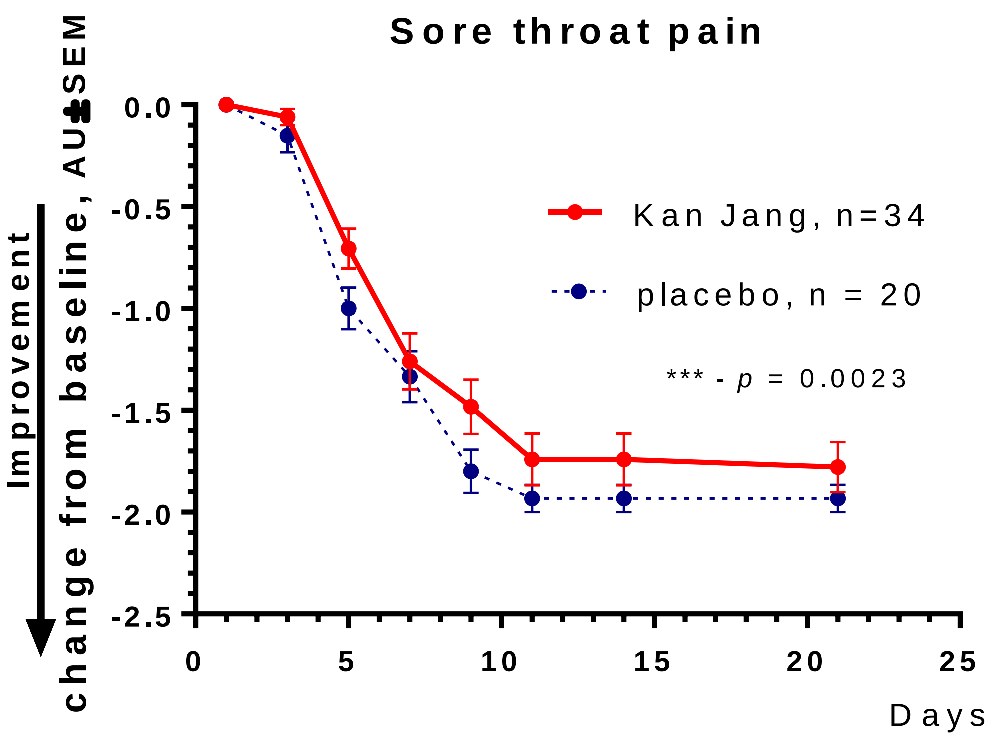

ITT analysis in a subgroup of 54 patients

Days	Kan Jang, n = 34			Placebo, n=20		
	Mean	SD	n	Mean	SD	n
1	0	0	34	0	0	20
3	-0,059	0,041	34	-0,150	0,082	20
5	-0,706	0,099	34	-1,000	0,103	20
7	-1,259	0,137	27	-1,333	0,126	15
9	-1,481	0,135	27	-1,800	0,107	15
11	-1,741	0,126	27	-1,933	0,067	15
14	-1,741	0,126	27	-1,933	0,067	15
21	-1,778	0,123	27	-1,933	0,067	15

Sore throat pain

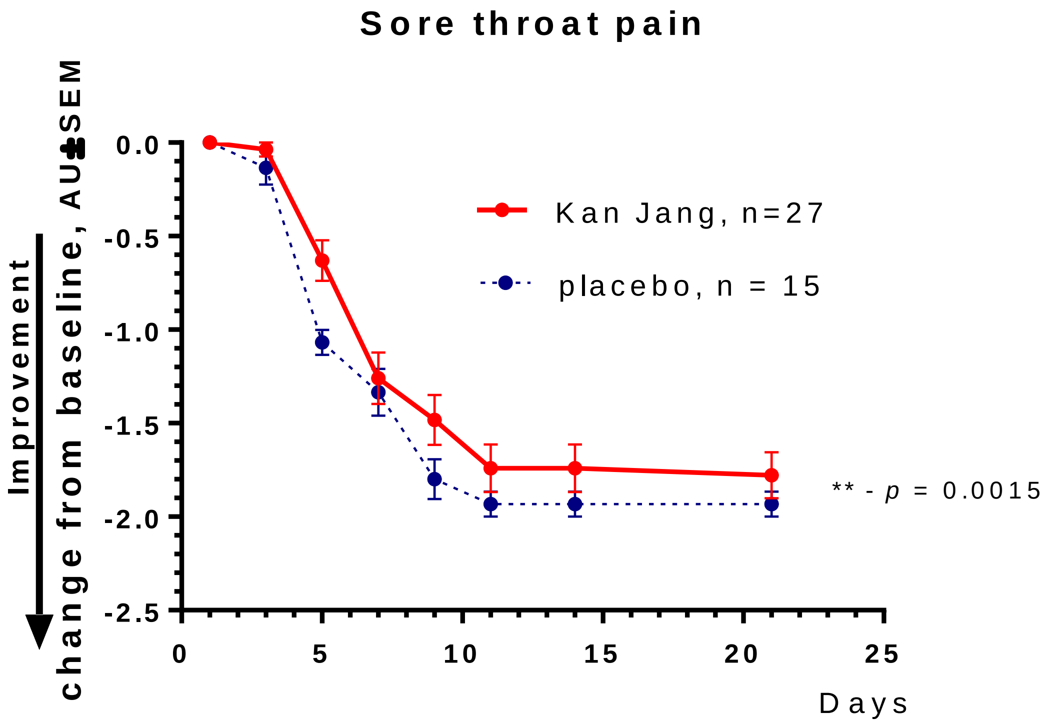

PP analysis in a subgroup of 42 patients

Days	Kan Jang, n = 27			Placebo, n=15		
	Mean	SD	n	Mean	SD	n
1	0	0	27	0	0	15
3	0	0	27	0	0	15
5	-0,037	0,037	27	0	0	15
7	-0,407	0,096	27	-0,133	0,091	15
9	-0,815	0,076	27	-0,667	0,126	15
11	-0,889	0,097	27	-0,667	0,126	15
14	-1,370	0,152	27	-1,067	0,228	15
21	-1,667	0,151	27	-1,333	0,252	15

Runny nose

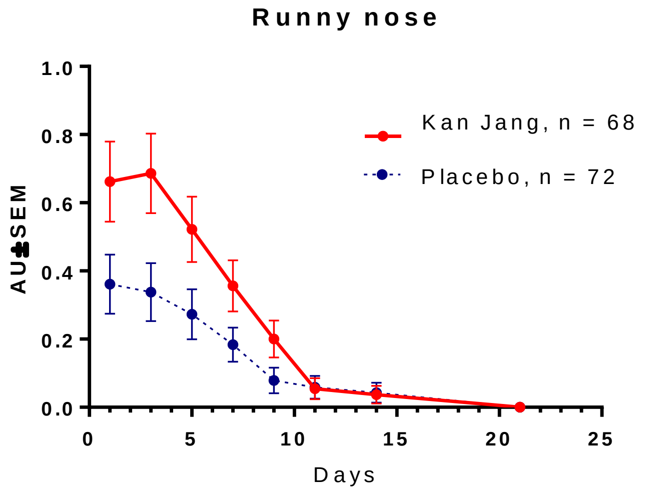

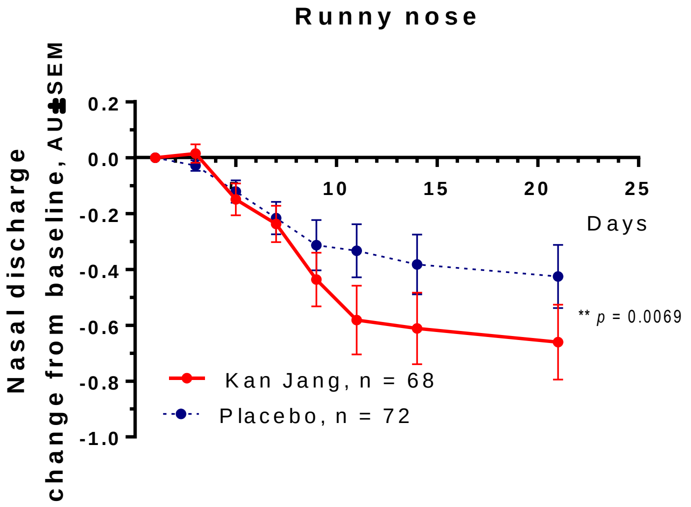

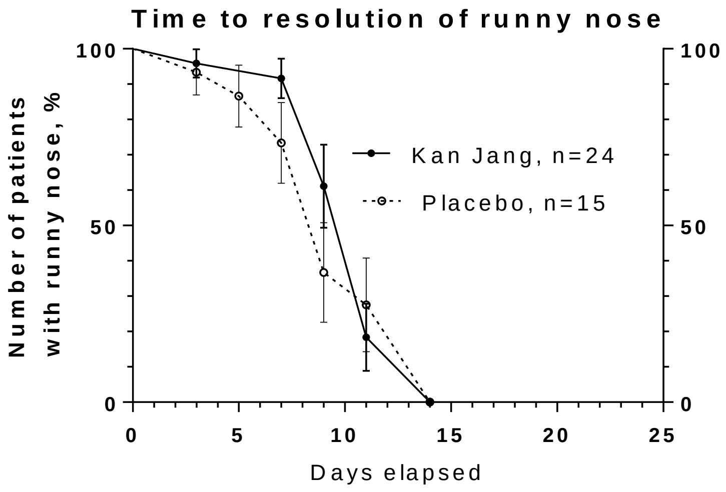

ITT analysis in 140 patients

Days	Kan Jang, n = 68			Placebo, n=72		
	Mean	SD	n	Mean	SD	n
1	0	0	68	0	0	72
3	0,015	0,034	67	-0,028	0,020	71
5	-0,149	0,057	67	-0,121	0,040	66
7	-0,237	0,065	59	-0,217	0,059	60
9	-0,436	0,096	55	-0,314	0,091	51
11	-0,582	0,124	55	-0,333	0,096	51
14	-0,611	0,128	54	-0,383	0,108	47
21	-0,660	0,135	53	-0,426	0,113	47

Runny nose PP analysis in 100 patients

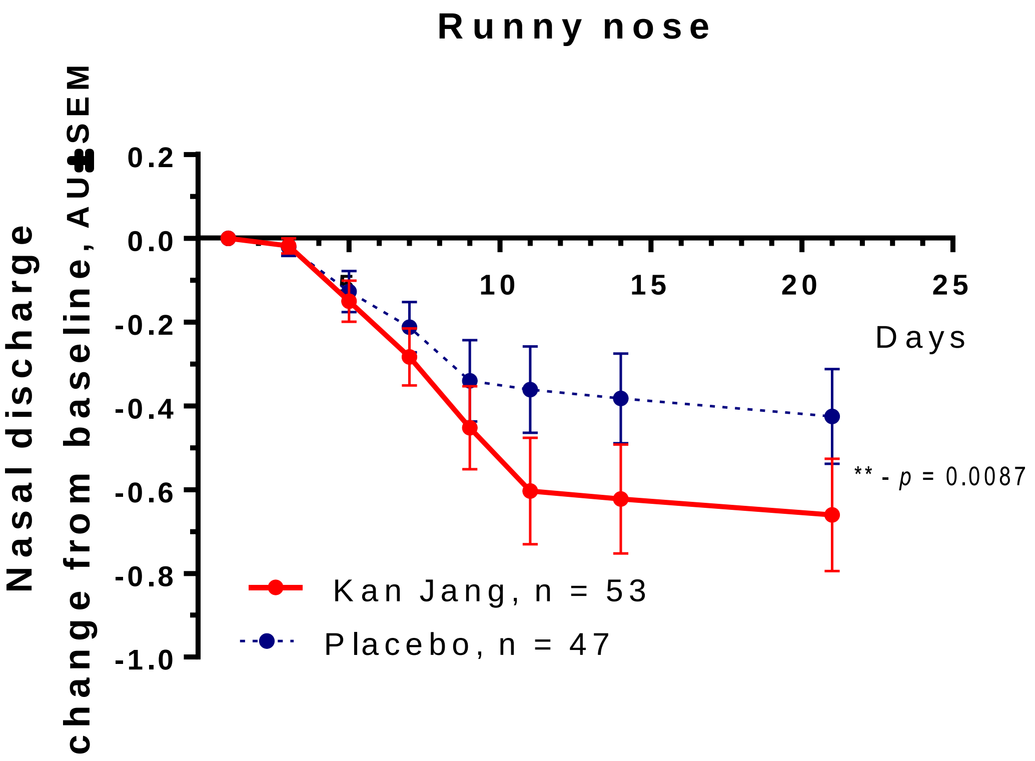

PP analysis in 100 patients

Days	Kan Jang, n = 53			Placebo, n=47		
	Mean	SD	n	Mean	SD	n
1	0	0	53	0	0	47
3	-0,019	0,019	53	-0,021	0,021	47
5	-0,151	0,050	53	-0,128	0,049	47
7	-0,283	0,068	53	-0,213	0,060	47
9	-0,453	0,099	53	-0,340	0,098	47
11	-0,604	0,127	53	-0,362	0,103	47
14	-0,623	0,130	53	-0,383	0,108	47
21	-0,660	0,135	53	-0,426	0,113	47

Runny nose

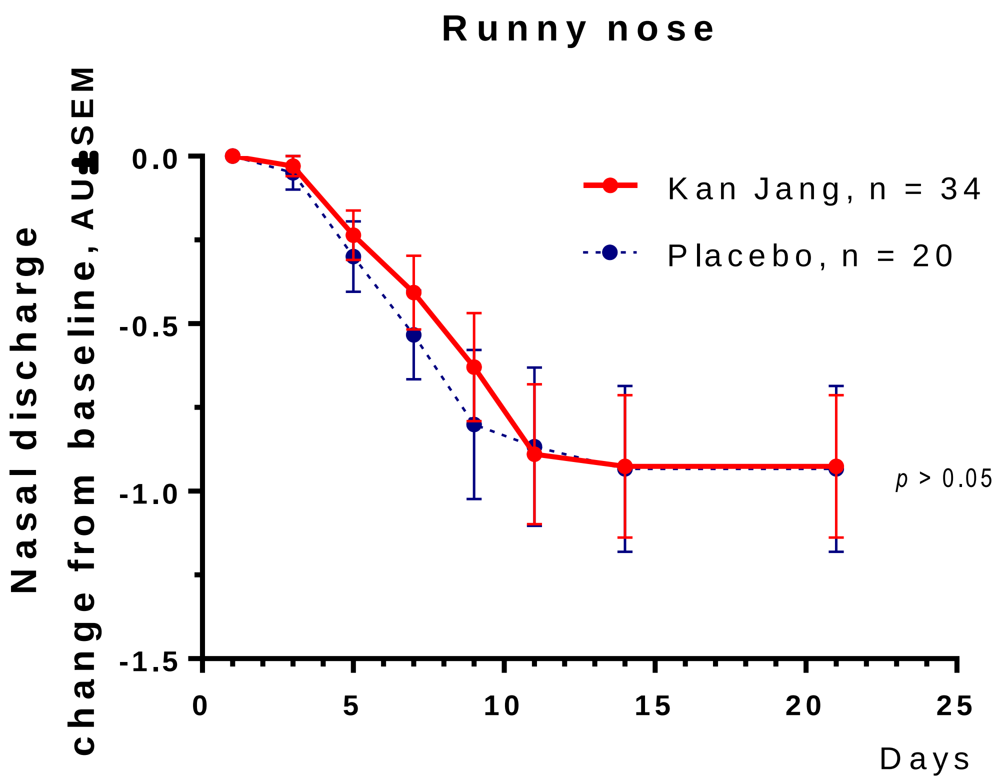

ITT analysis in a subgroup of 54 patients

Days	Kan Jang, n = 34			Placebo, n=20		
	Mean	SD	n	Mean	SD	n
1	0	0	34	0	0	20
3	-0,029	0,029	34,000	-0,050	0,050	20
5	-0,235	0,074	34,000	-0,300	0,105	20
7	-0,407	0,110	27,000	-0,533	0,133	15
9	-0,630	0,161	27,000	-0,800	0,223	15
11	-0,889	0,209	27,000	-0,867	0,236	15
14	-0,926	0,213	27,000	-0,933	0,248	15
21	-0,926	0,213	27,000	-0,933	0,248	15

Runny nose

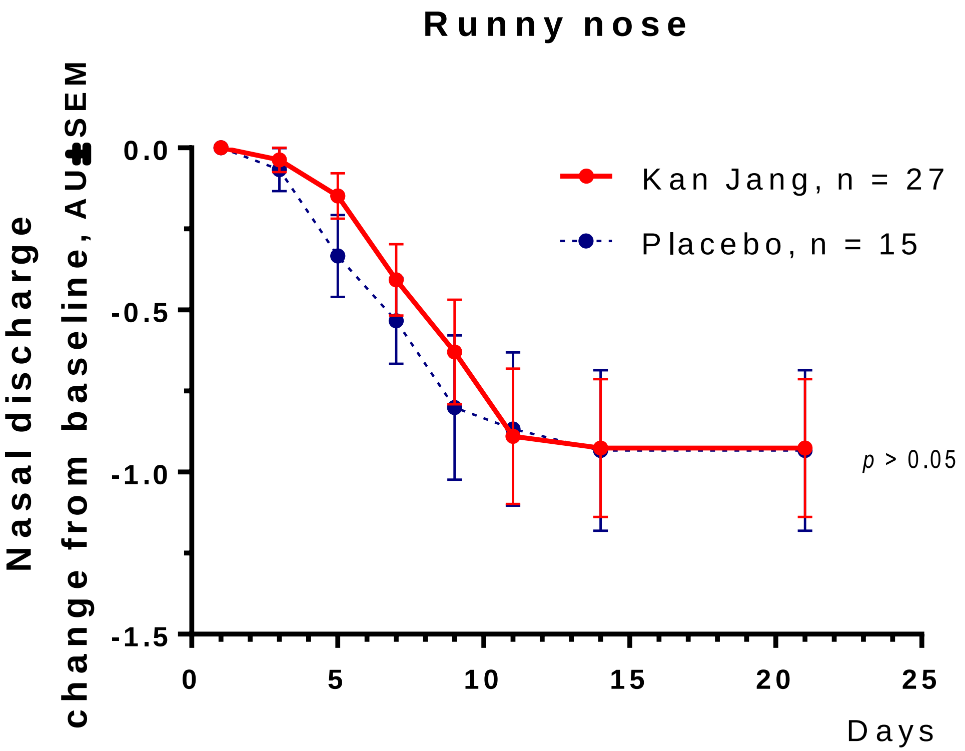

PP analysis in a subgroup of 42 patients

Days	Kan Jang, n = 27			Placebo, n=15		
	Mean	SD	n	Mean	SD	n
1	0	0	27	0	0	15
3	-0,037	0,037	27	-0,067	0,067	15
5	-0,148	0,070	27	-0,333	0,126	15
7	-0,407	0,110	27	-0,533	0,133	15
9	-0,630	0,161	27	-0,800	0,223	15
11	-0,889	0,209	27	-0,867	0,236	15
14	-0,926	0,213	27	-0,933	0,248	15
21	-0,926	0,213	27	-0,933	0,248	15

Muscle soreness

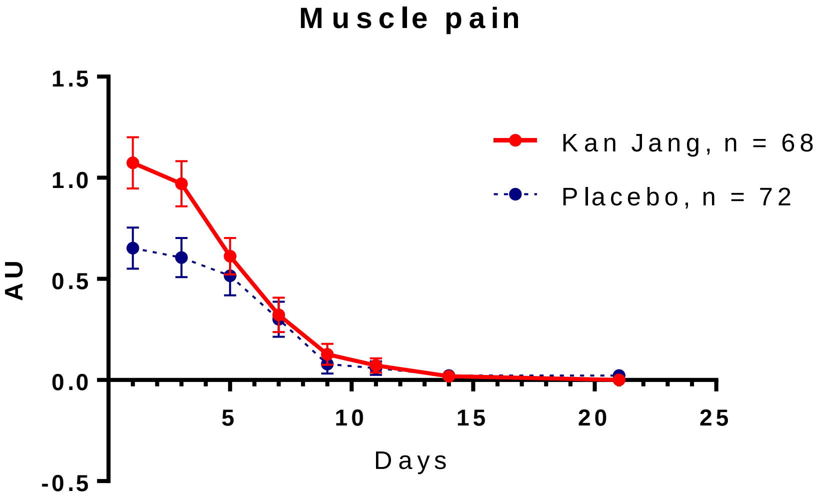

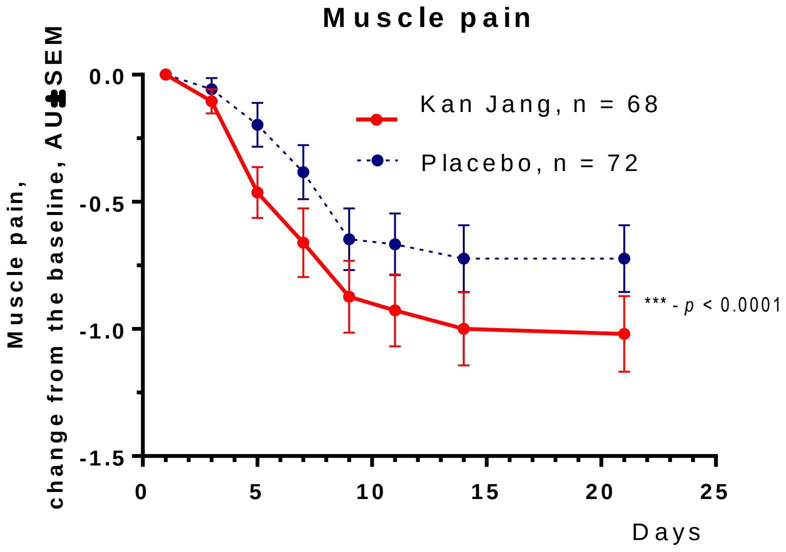

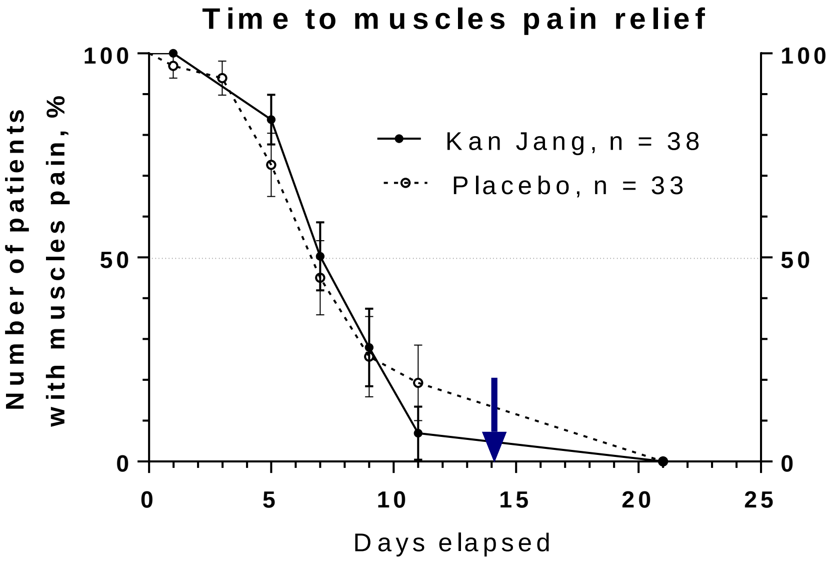

ITT analysis in 140 patients

Days	Kan Jang, n = 68			Placebo, n=72		
	Mean	SD	n	Mean	SD	n
1	0,000	0,000	68	0	0	72
3	-0,104	0,048	67	-0,056	0,044	71
5	-0,463	0,100	67	-0,197	0,087	66
7	-0,661	0,136	59	-0,383	0,107	60
9	-0,873	0,142	55	-0,647	0,122	51
11	-0,927	0,142	55	-0,667	0,121	51
14	-1,000	0,145	54	-0,723	0,132	47
21	-1,019	0,149	53	-0,723	0,132	47

Muscle soreness

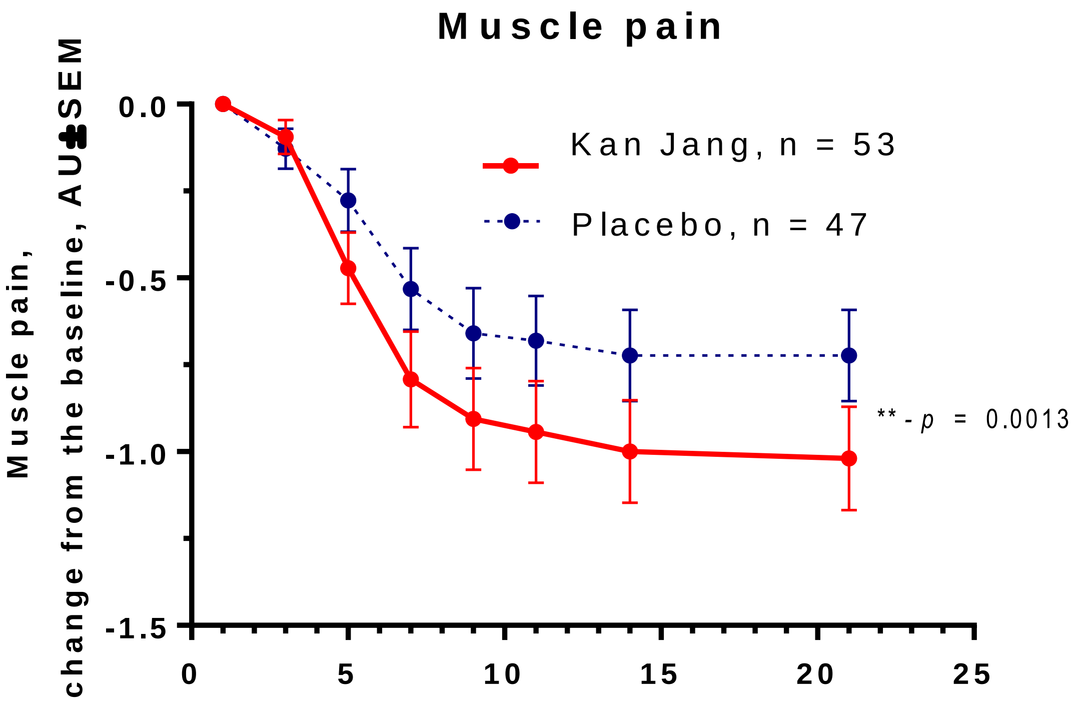

PP analysis in 100 patients

Days	Kan Jang, n = 53			Placebo, n=47		
	Mean	SD	n	Mean	SD	n
1	0	0	53	0	0	47
3	-0,094	0,049	53	-0,128	0,058	47
5	-0,472	0,103	53	-0,277	0,090	47
7	-0,792	0,138	53	-0,532	0,117	47
9	-0,906	0,146	53	-0,660	0,130	47
11	-0,943	0,146	53	-0,681	0,129	47
14	-1,000	0,148	53	-0,723	0,132	47
21	-1,019	0,149	53	-0,723	0,132	47

Muscle soreness

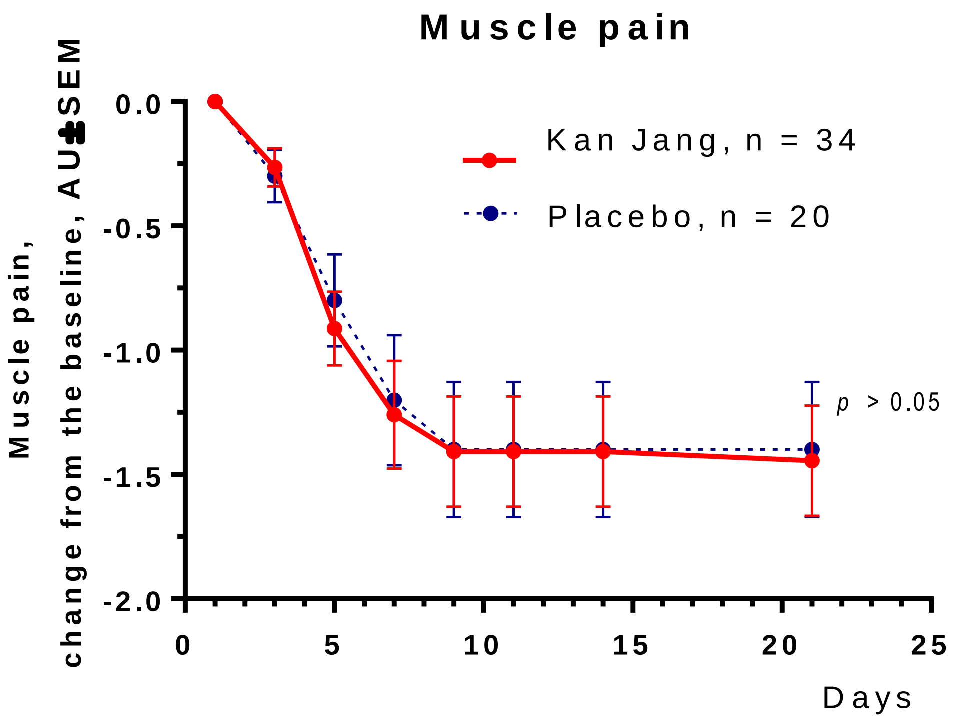

ITT analysis in a subgroup of 54 patients

Days	Kan Jang, n = 34			Placebo, n=20		
	Mean	SD	n	Mean	SD	n
1	0	0	34	0	0	20
3	-0,029	0,029	34,000	-0,050	0,050	20
5	-0,235	0,074	34,000	-0,300	0,105	20
7	-0,407	0,110	27,000	-0,533	0,133	15
9	-0,630	0,161	27,000	-0,800	0,223	15
11	-0,889	0,209	27,000	-0,867	0,236	15
14	-0,926	0,213	27,000	-0,933	0,248	15
21	-0,926	0,213	27,000	-0,933	0,248	15

Muscle soreness

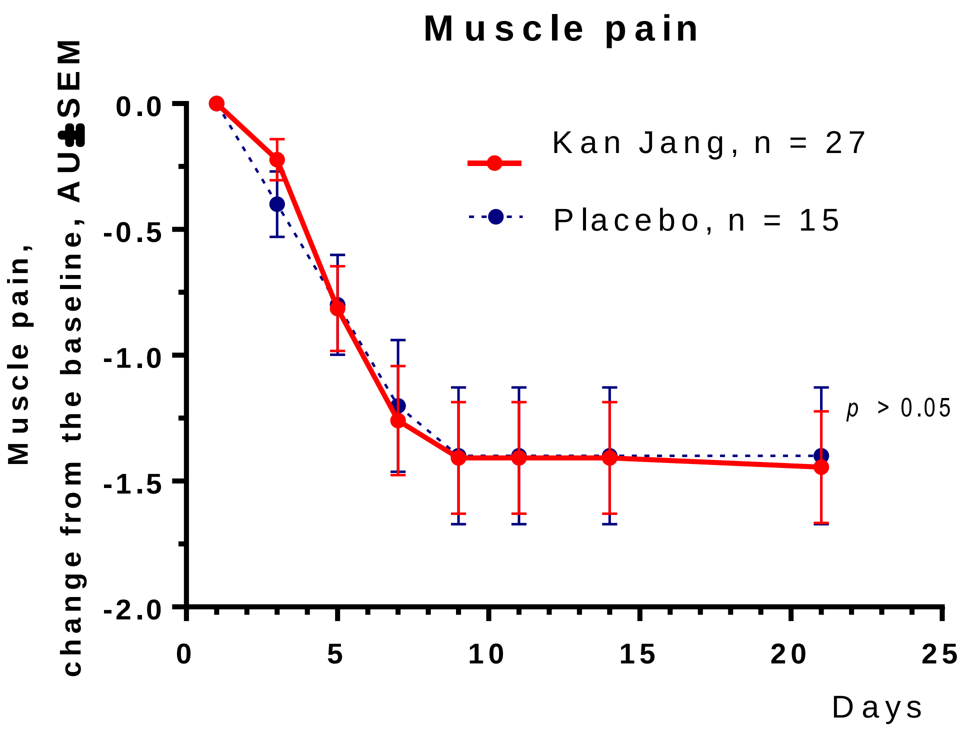

PP analysis in a subgroup of 42 patients

Days	Kan Jang, n = 27			Placebo, n=15		
	Mean	SD	n	Mean	SD	n
1	0	0	27	0	0	15
3	-0,222	0,082	27	-0,400	0,131	15
5	-0,815	0,169	27	-0,800	0,200	15
7	-1,259	0,217	27	-1,200	0,262	15
9	-1,407	0,222	27	-1,400	0,273	15
11	-1,407	0,222	27	-1,400	0,273	15
14	-1,407	0,222	27	-1,400	0,273	15
21	-1,444	0,222	27	-1,400	0,273	15

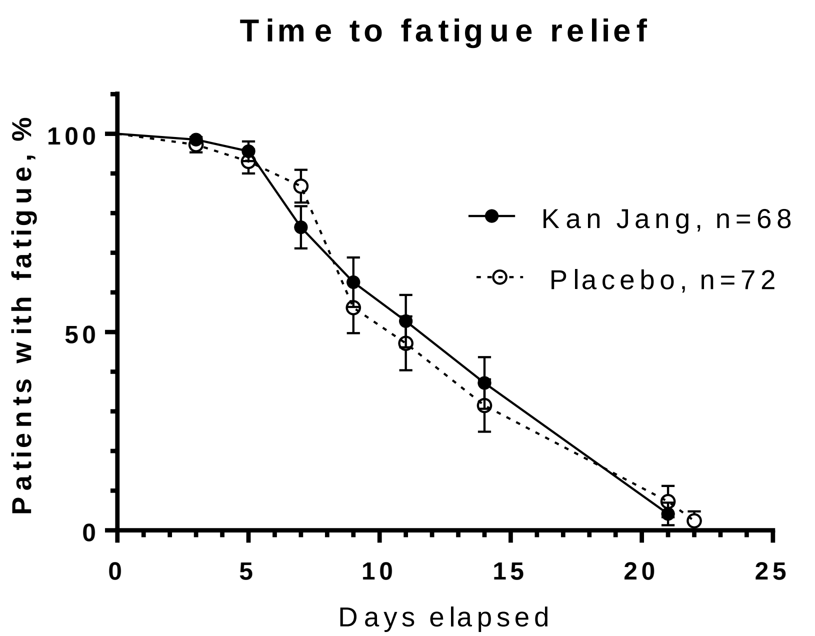

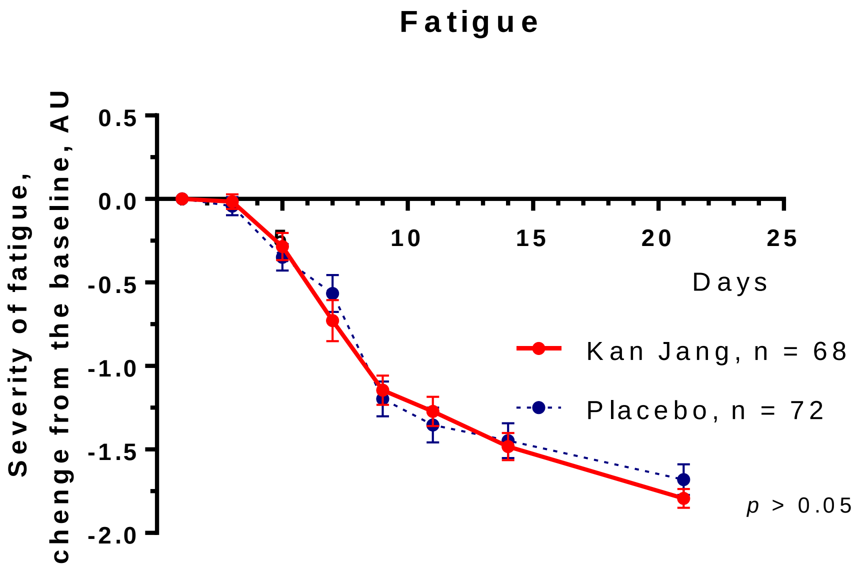

Fatigue

ITT analysis in 140 patients

Days	Kan Jang, n = 68			Placebo, n=72		
	Mean	SD	n	Mean	SD	n
1	0	0	68	0	0	72
3	-0,015	0,045	67	-0,042	0,055	71
5	-0,284	0,082	67	-0,348	0,079	66
7	-0,729	0,123	59	-0,567	0,112	60
9	-1,145	0,088	55	-1,196	0,105	51
11	-1,273	0,088	55	-1,353	0,104	51
14	-1,481	0,083	54	-1,447	0,105	47
21	-1,792	0,056	53	-1,681	0,092	47

Fatigue

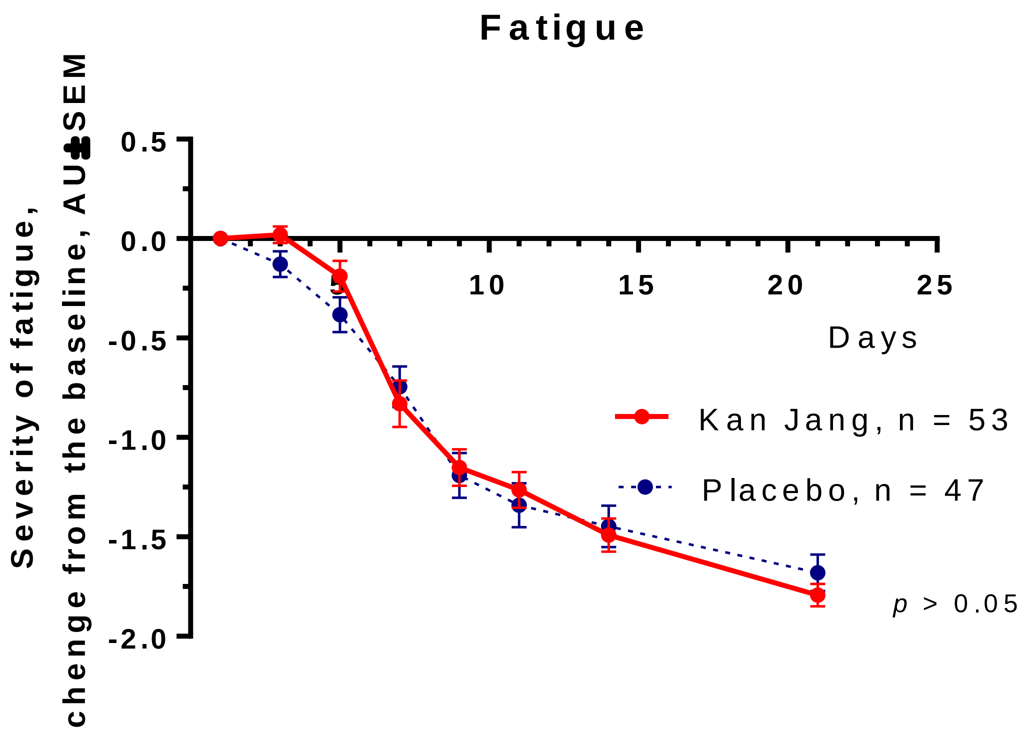

PP analysis in 100 patients

Days	Kan Jang, n = 53			Placebo, n=47		
	Mean	SD	n	Mean	SD	n
1	0	0	53	0	0	47
3	0,019	0,043	53	-0,128	0,065	47
5	-0,189	0,076	53	-0,383	0,089	47
7	-0,830	0,117	53	-0,745	0,103	47
9	-1,151	0,091	53	-1,191	0,112	47
11	-1,264	0,090	53	-1,340	0,111	47
14	-1,491	0,084	53	-1,447	0,105	47
21	-1,792	0,056	53	-1,681	0,092	47

Fatigue

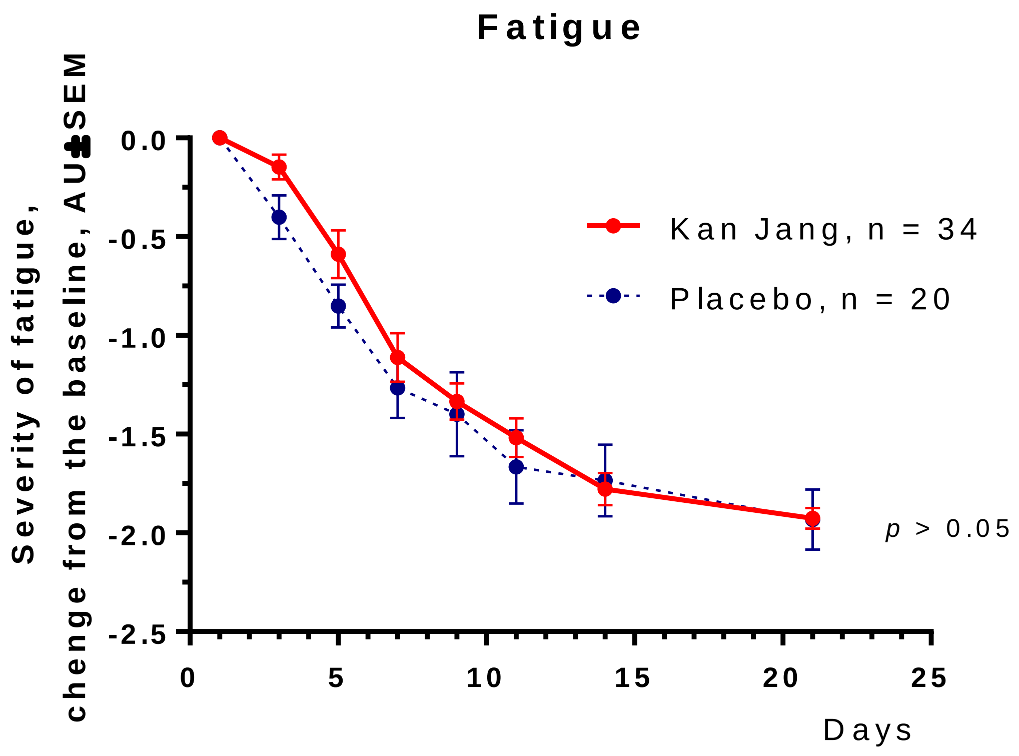

ITT analysis in a subgroup of 54 patients

Days	Kan Jang, n = 34			Placebo, n=20		
	Mean	SD	n	Mean	SD	n
1	0	0	34	0	0	20
3	-0,147	0,062	34	-0,4	0,11239	20
5	-0,588	0,120	34	-0,85	0,109424	20
7	-1,111	0,123	27	-1,26667	0,153271	15
9	-1,333	0,092	27	-1,4	0,213809	15
11	-1,519	0,098	27	-1,66667	0,186871	15
14	-1,778	0,082	27	-1,73333	0,181703	15
21	-1,926	0,051	27	-1,93333	0,153271	15

Fatigue

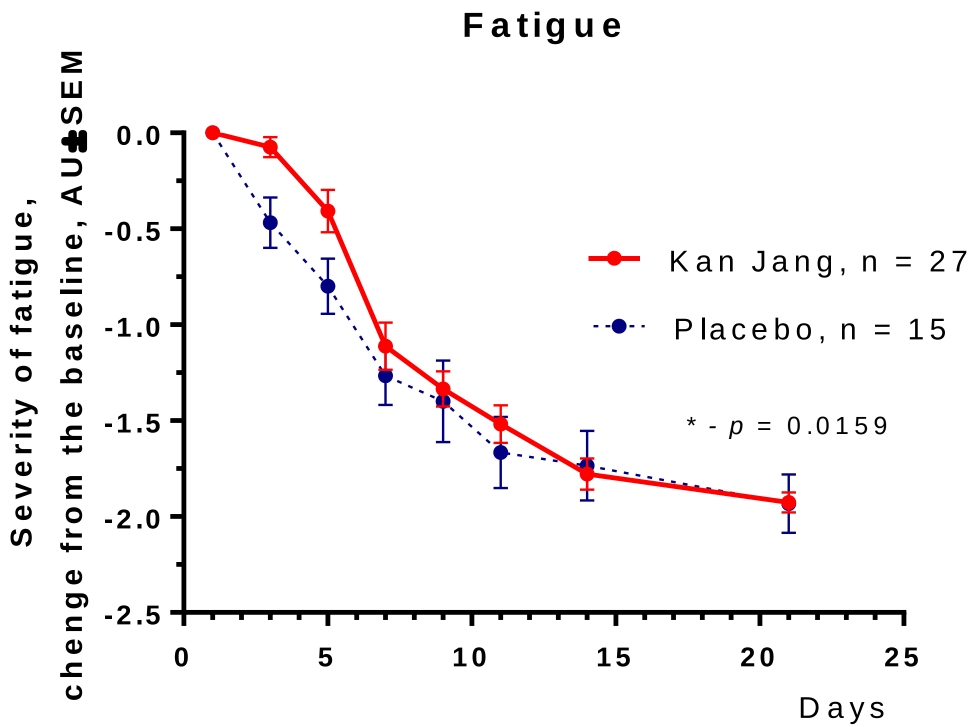

PP analysis in a subgroup of 42 patients

Days	Kan Jang, n = 27			Placebo, n=15		
	Mean	SD	n	Mean	SD	n
1	0	0	27	0	0	15
3	-0,07	0,05	27	-0,47	0,13	15
5	-0,41	0,11	27	-0,80	0,14	15
7	-1,11	0,12	27	-1,27	0,15	15
9	-1,33	0,09	27	-1,40	0,21	15
11	-1,52	0,10	27	-1,67	0,19	15
14	-1,78	0,08	27	-1,73	0,18	15
21	-1,93	0,05	27	-1,93	0,15	15

Headache

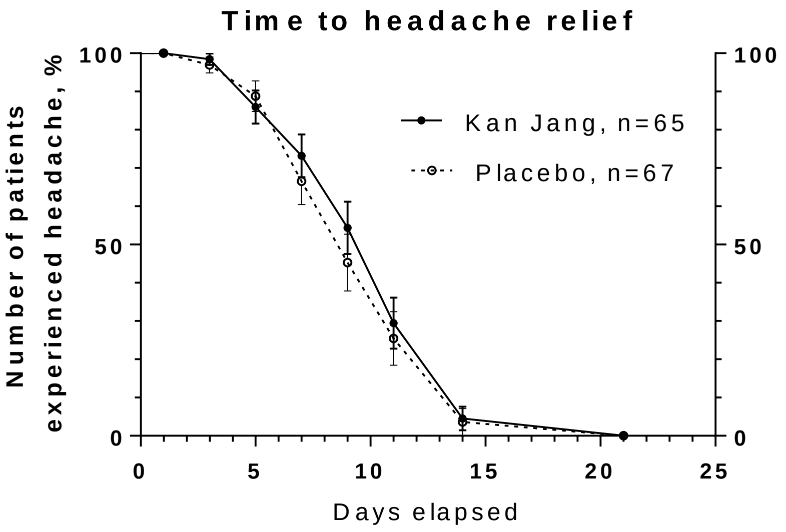

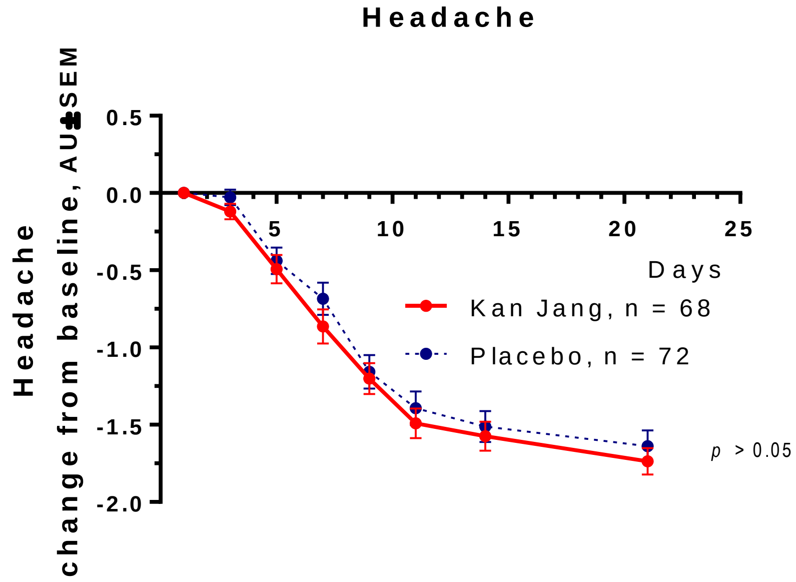

ITT analysis in 140 patients

Days	Kan Jang, n = 68			Placebo, n=72		
	Mean	SD	n	Mean	SD	n
1	0	0	68	0	0	72
3	-0,119	0,050	67	-0,028	0,049	71
5	-0,493	0,091	67	-0,439	0,087	66
7	-0,864	0,112	59	-0,683	0,105	60
9	-1,200	0,102	55	-1,157	0,110	51
11	-1,491	0,097	55	-1,392	0,109	51
14	-1,574	0,094	54	-1,511	0,100	47
21	-1,736	0,086	53	-1,638	0,103	47

Headache

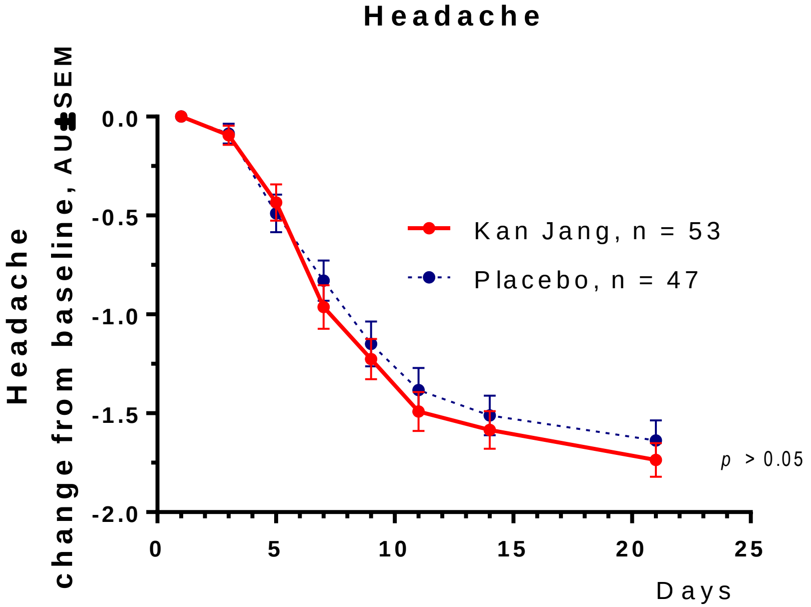

PP analysis in 100 patients

Days	Kan Jang, n = 53			Placebo, n=47		
	Mean	SD	n	Mean	SD	n
1	0	0	53	0	0	47
3	-0,094	0,049	53	-0,085	0,051	47
5	-0,434	0,091	53	-0,489	0,096	47
7	-0,962	0,111	53	-0,830	0,102	47
9	-1,226	0,103	53	-1,149	0,114	47
11	-1,491	0,099	53	-1,383	0,112	47
14	-1,585	0,095	53	-1,511	0,100	47
21	-1,736	0,086	53	-1,638	0,103	47

Headache

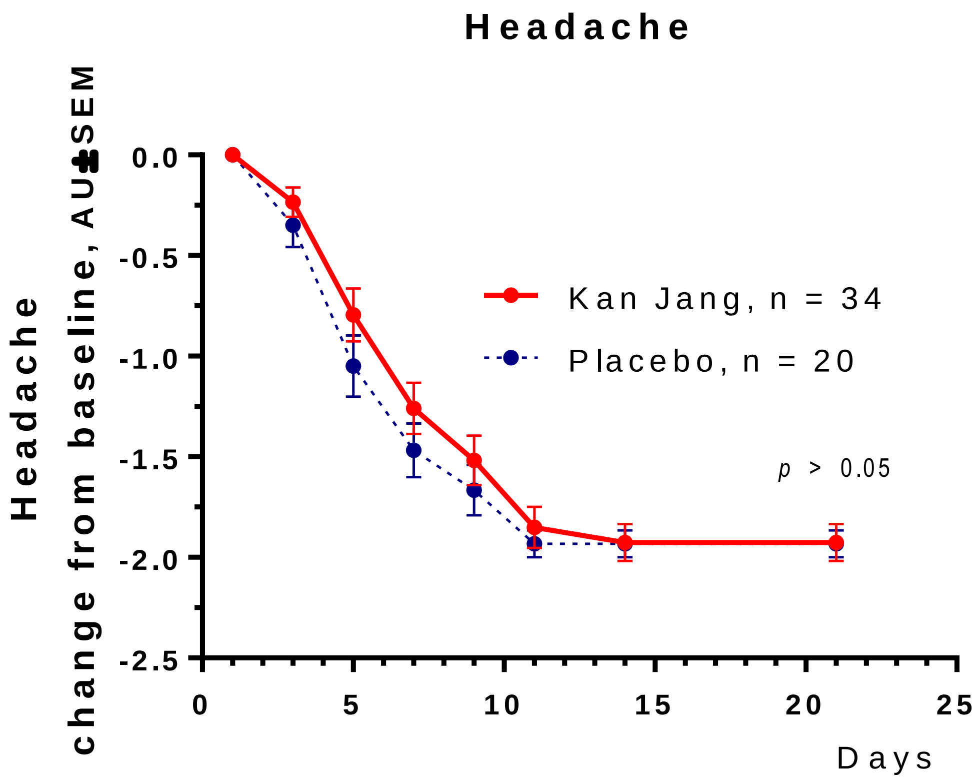

ITT analysis in a subgroup of 54 patients

Days	Kan Jang, n = 34			Placebo, n=20		
	Mean	SD	n	Mean	SD	n
1	0	0	34	0	0	20
3	-0,235	0,074	34	-0,350	0,109	20
5	-0,794	0,132	34	-1,050	0,153	20
7	-1,259	0,126	27	-1,467	0,133	15
9	-1,519	0,124	27	-1,667	0,126	15
11	-1,852	0,103	27	-1,933	0,067	15
14	-1,926	0,091	27	-1,933	0,067	15
21	-1,926	0,091	27	-1,933	0,067	15

Headache

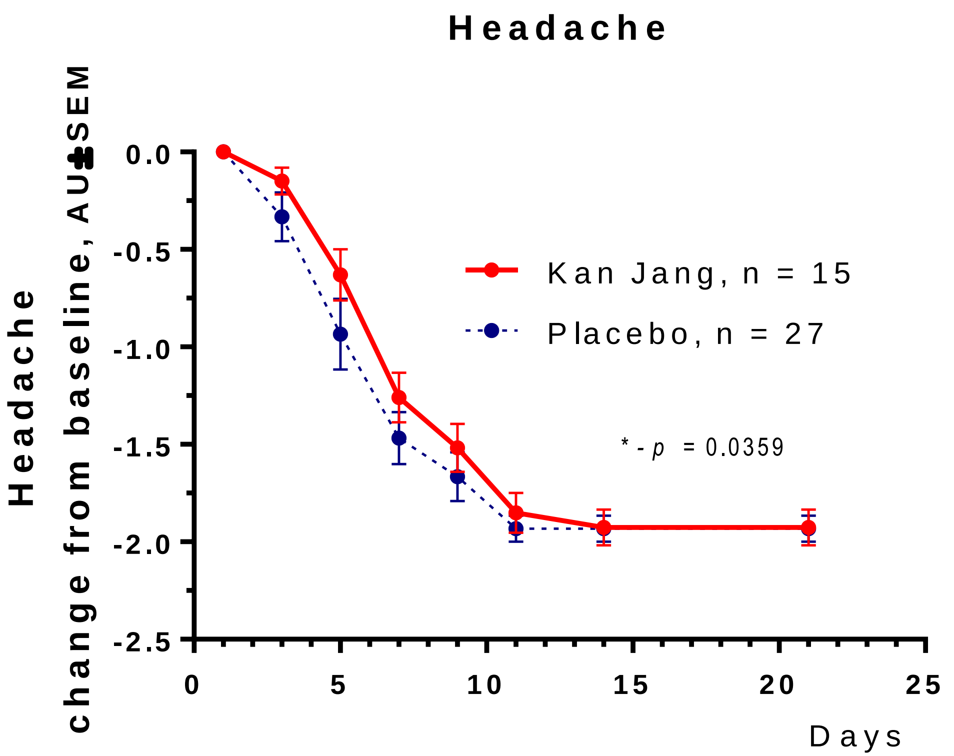

PP analysis in a subgroup of 42 patients

Days	Kan Jang, n = 27			Placebo, n=15		
	Mean	SD	n	Mean	SD	n
1	0	0	27	0	0	15
3	-0,148	0,070	27	-0,333	0,126	15
5	-0,630	0,132	27	-0,933	0,182	15
7	-1,259	0,126	27	-1,467	0,133	15
9	-1,519	0,124	27	-1,667	0,126	15
11	-1,852	0,103	27	-1,933	0,067	15
14	-1,926	0,091	27	-1,933	0,067	15
21	-1,926	0,091	27	-1,933	0,067	15

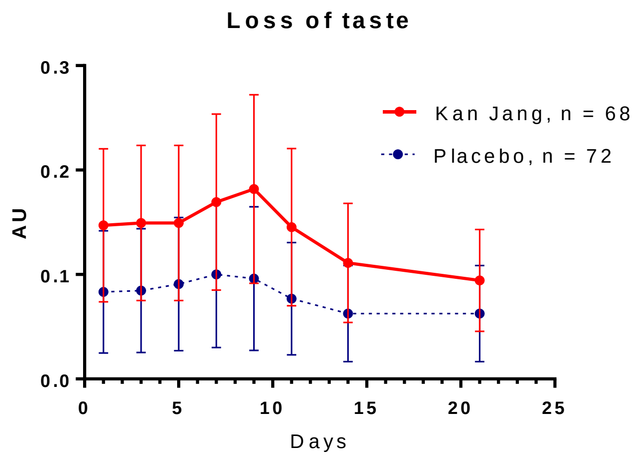

Loss of taste

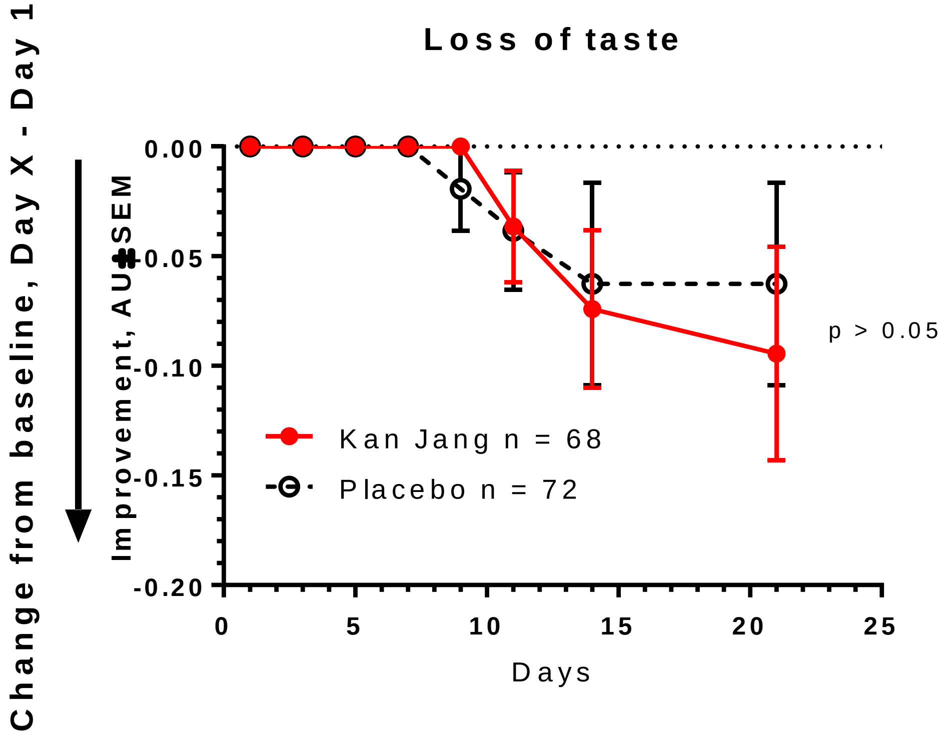

ITT analysis in 140 patients

Days	Kan Jang, n = 68			Placebo, n=72		
	Mean	SD	n	Mean	SD	n
1	0	0	68	0	0	72
3	0	0	67	0	0	71
5	0	0	67	0	0	66
7	0	0	59	0	0	60
9	0	0	55	-0,019	0,019	52
11	-0,036	0,025	55	-0,038	0,027	52
14	-0,074	0,036	54	-0,063	0,046	48
21	-0,094	0,049	53	-0,063	0,046	48

Loss of taste

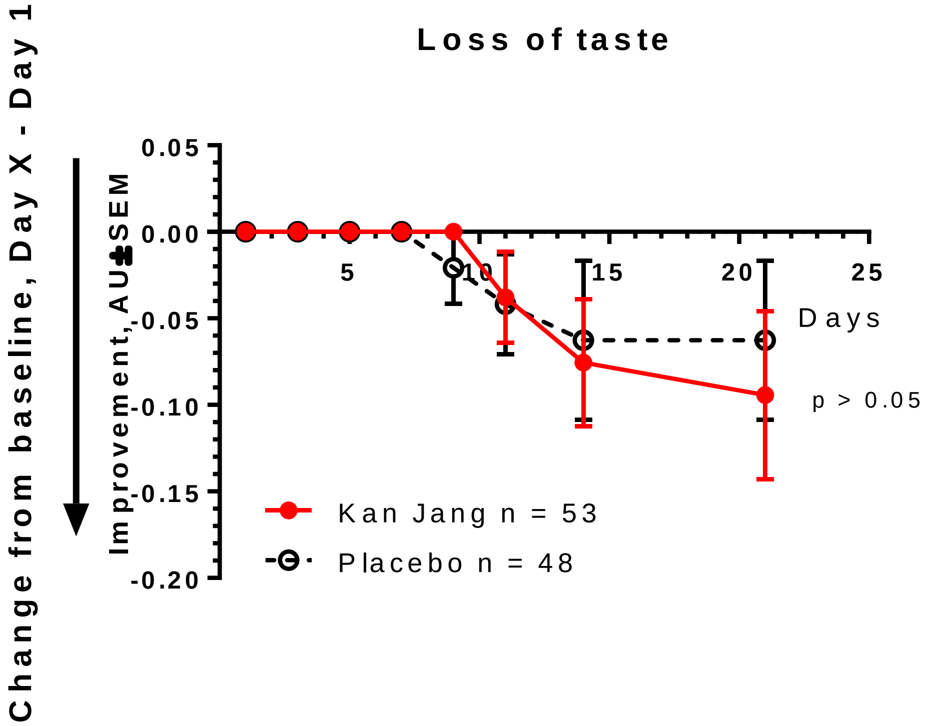

PP analysis in 101 patients

Days	Kan Jang, n = 53			Placebo, n=47		
	Mean	SD	n	Mean	SD	n
1	0	0	53	0	0	48
3	0	0	53	0	0	48
5	0	0	53	0	0	48
7	0	0	53	0	0	48
9	0	0	53	-0,021	0,021	48
11	-0,038	0,026	53	-0,042	0,029	48
14	-0,075	0,037	53	-0,063	0,046	48
21	-0,094	0,049	53	-0,063	0,046	48

Loss of taste

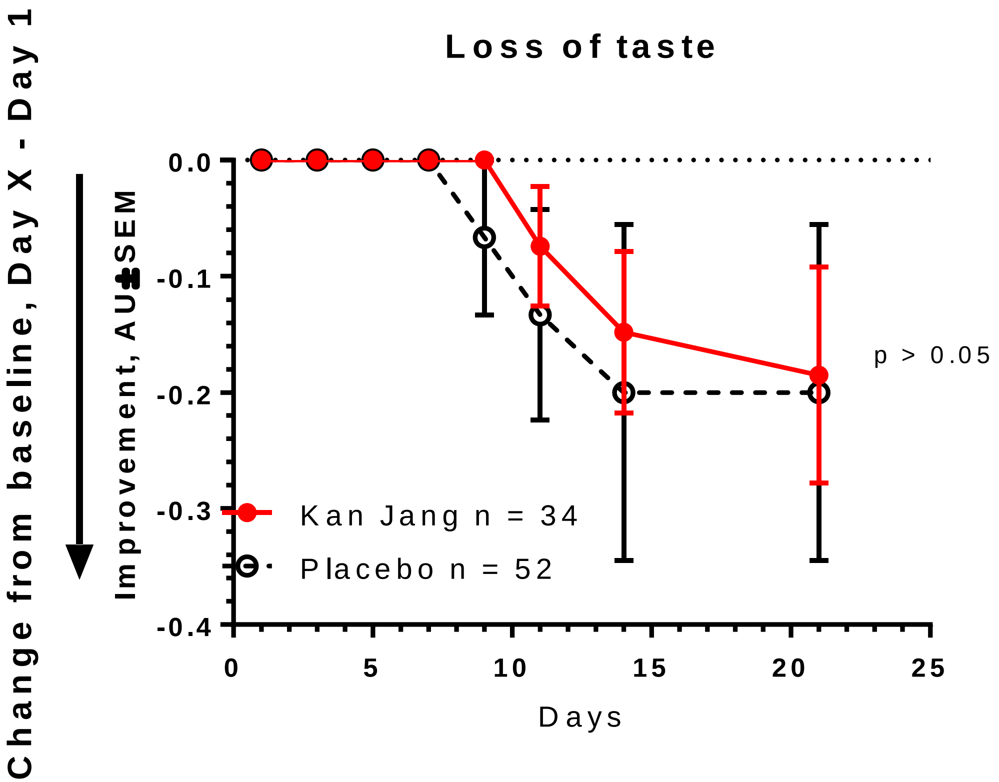

ITT analysis in a subgroup of 54 patients

Days	Kan Jang, n = 34			Placebo, n=20		
	Mean	SD	n	Mean	SD	n
1	0	0	34	0	0	20
3	0	0	34	0	0	20
5	0	0	34	0	0	20
7	0	0	27	0	0	15
9	0	0	27	-0,067	0,067	15
11	-0,074	0,051	27	-0,133	0,091	15
14	-0,148	0,070	27	-0,200	0,145	15
21	-0,185	0,093	27	-0,200	0,145	15

Loss of taste

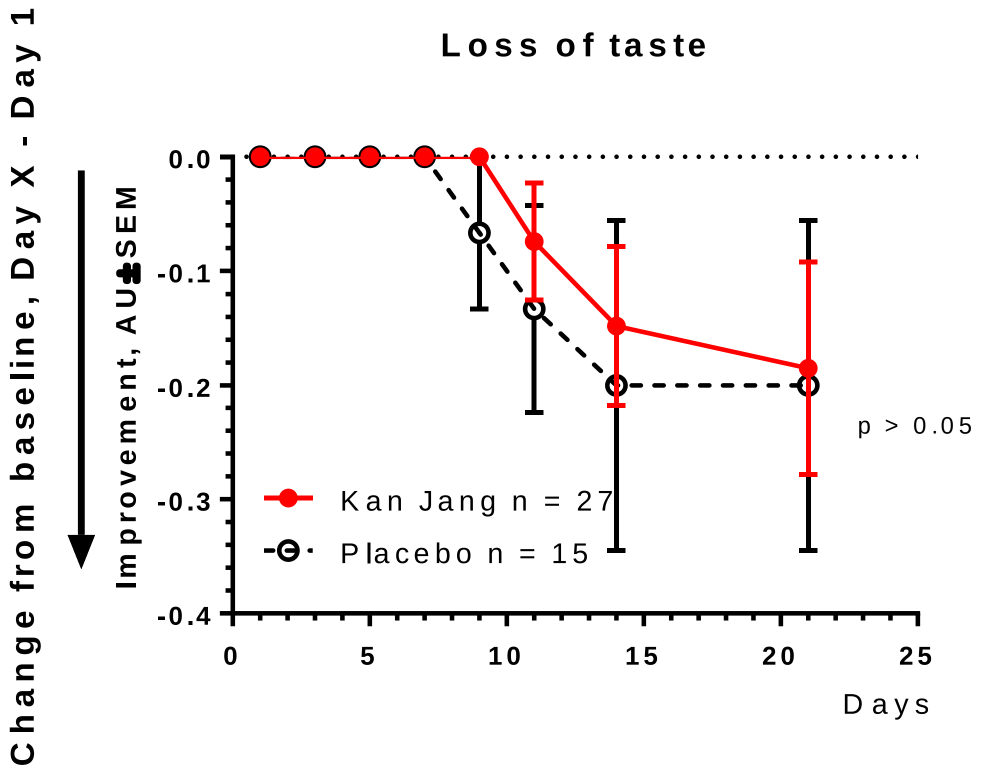

PP analysis in a subgroup of 42 patients

Days	Kan Jang, n = 27			Placebo, n=15		
	Mean	SD	n	Mean	SD	n
1	0	0	27	0	0	15
3	0	0	27	0	0	15
5	0	0	27	0	0	15
7	0	0	27	0	0	15
9	0	0	27	-0,067	0,067	15
11	-0,074	0,051	27	-0,133	0,091	15
14	-0,148	0,070	27	-0,200	0,145	15
21	-0,185	0,093	27	-0,200	0,145	15

Loss of smell

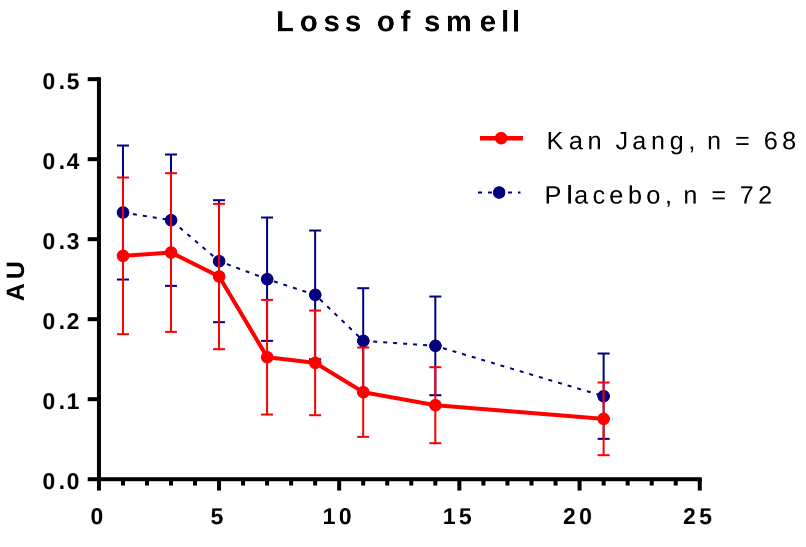

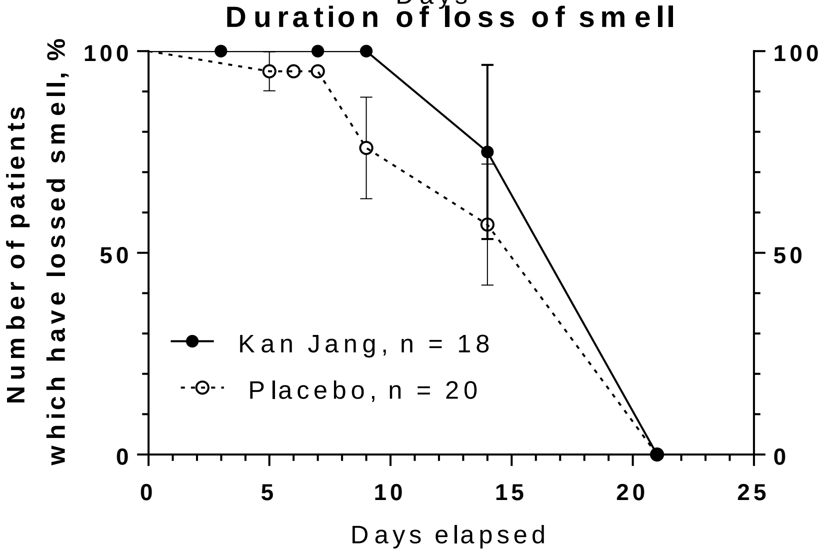

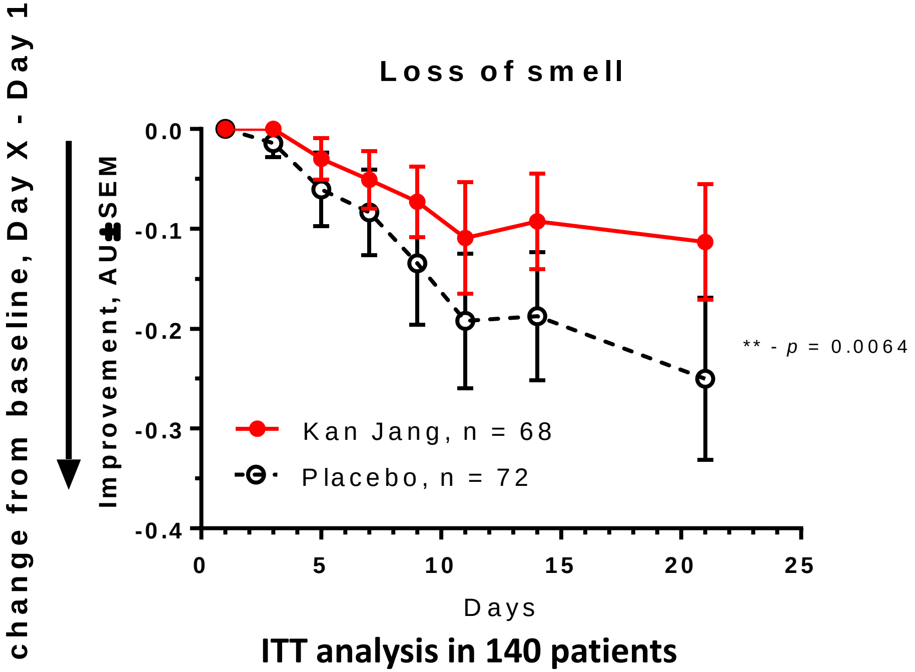

ITT analysis in 140 patients

Days	Kan Jang, n = 68			Placebo, n=72		
	Mean	SD	n	Mean	SD	n
1	0	0	68	0	0	72
3	0	0	67	-0,01	0,01	71
5	-0,03	0,02	67	-0,06	0,04	66
7	-0,05	0,03	59	-0,08	0,04	60
9	-0,07	0,04	55	-0,13	0,06	52
11	-0,11	0,06	55	-0,19	0,07	52
14	-0,09	0,05	54	-0,19	0,06	48
21	-0,11	0,06	53	-0,25	0,08	48

Loss of smell

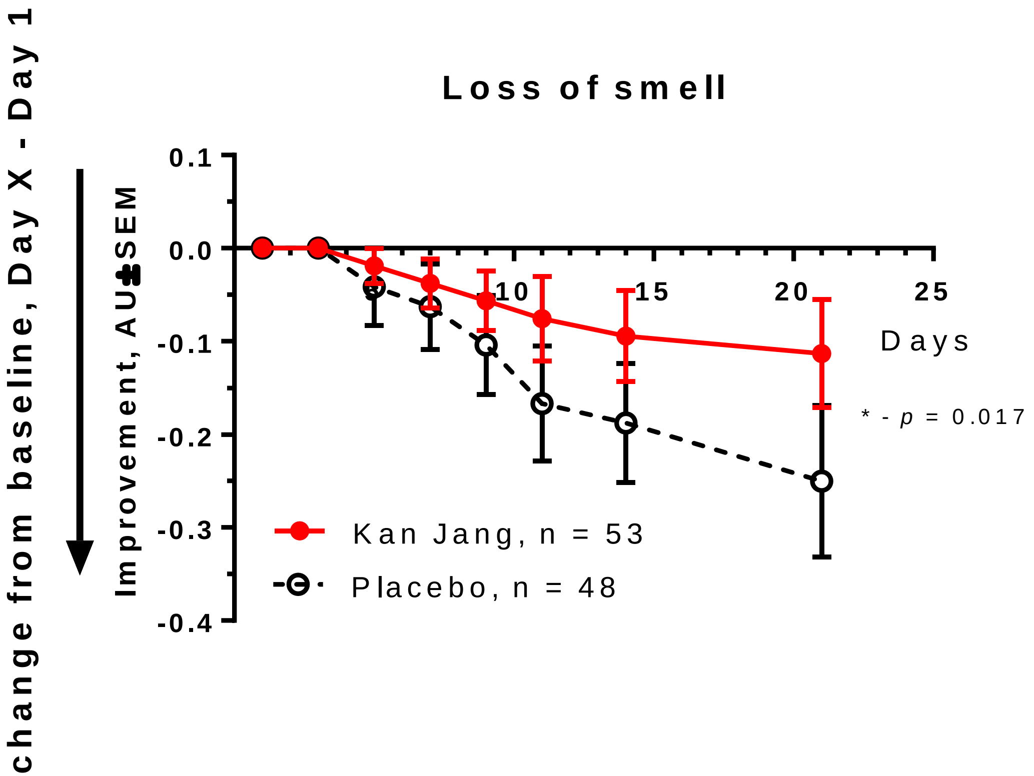

PP analysis in 101 patients

Days	Kan Jang, n = 53			Placebo, n=47		
	Mean	SD	n	Mean	SD	n
1	0	0	53	0	0	48
3	0	0	53	0	0	48
5	-0,019	0,019	53	-0,042	0,042	48
7	-0,038	0,026	53	-0,063	0,046	48
9	-0,057	0,032	53	-0,104	0,054	48
11	-0,075	0,045	53	-0,167	0,062	48
14	-0,094	0,049	53	-0,188	0,064	48
21	-0,113	0,058	53	-0,250	0,082	48

Loss of smell

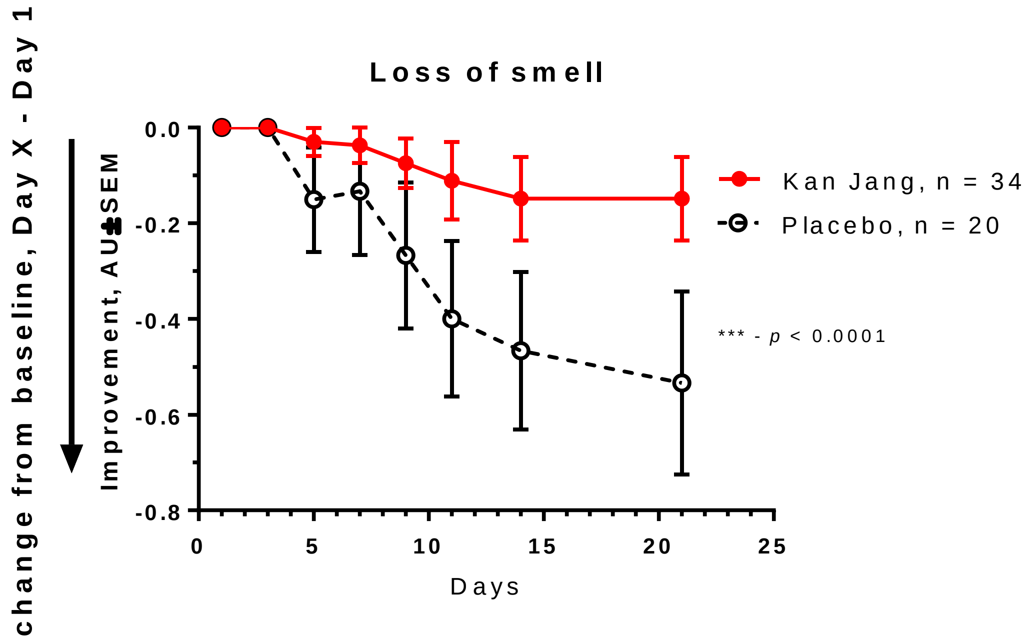

ITT analysis in a subgroup of 54 patients

Days	Kan Jang, n = 34			Placebo, n=20		
	Mean	SD	n	Mean	SD	n
1	0	0	34	0	0	20
3	0	0	34	0	0	20
5	-0,029	0,029	34	-0,150	0,109	20
7	-0,037	0,037	27	-0,133	0,133	15
9	-0,074	0,051	27	-0,267	0,153	15
11	-0,111	0,082	27	-0,400	0,163	15
14	-0,148	0,088	27	-0,467	0,165	15
21	-0,148	0,088	27	-0,533	0,192	15

Loss of smell

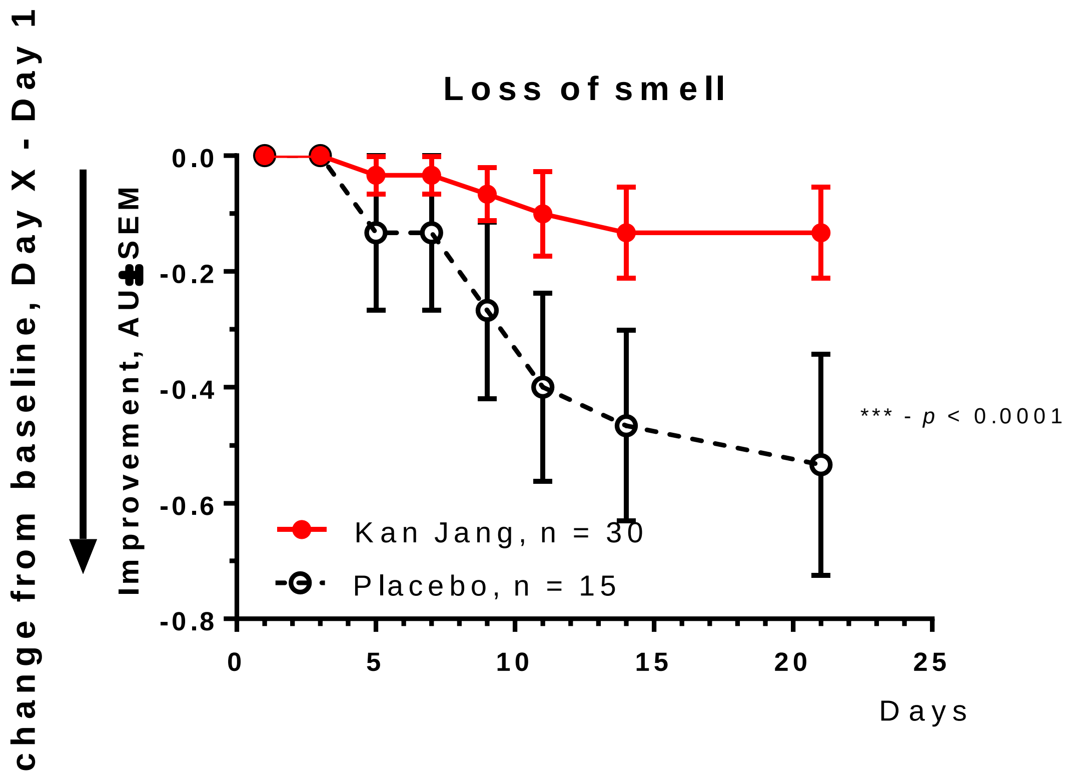

PP analysis in a subgroup of 45 patients

Days	Kan Jang, n = 27			Placebo, n=15		
	Mean	SD	n	Mean	SD	n
1	0	0	30	0	0	15
3	0	0	30	0	0	15
5	-0,033	0,033	30	-0,13	0,13	15
7	-0,033	0,033	30	-0,13	0,13	15
9	-0,067	0,046	30	-0,27	0,15	15
11	-0,100	0,074	30	-0,40	0,16	15
14	-0,133	0,079	30	-0,47	0,17	15
21	-0,133	0,079	30	-0,53	0,19	15

Physical activity

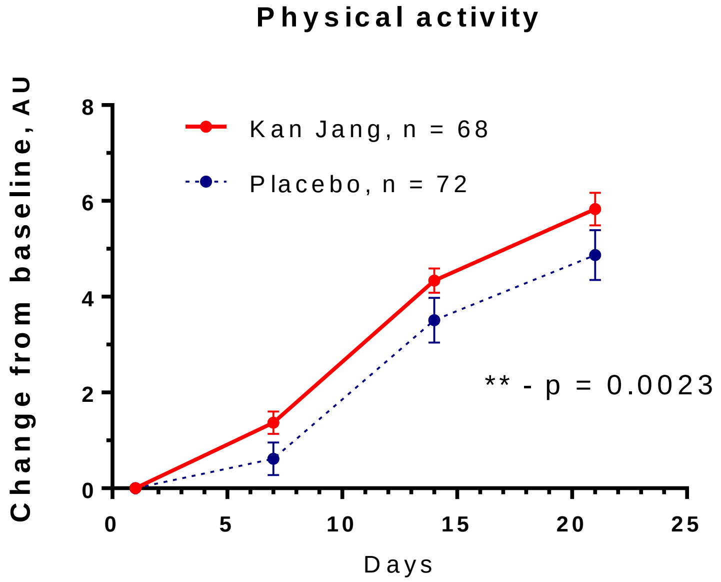

ITT analysis in 140 patients

Days	Kan Jang, n = 68			Placebo, n=72		
	Mean	SD	n	Mean	SD	n
1	0	0	68	0	0	72
7	1,373	0,239	59	0,617	0,341	60
14	4,333	0,254	54	3,511	0,467	47
21	5,830	0,343	53	4,872	0,523	47

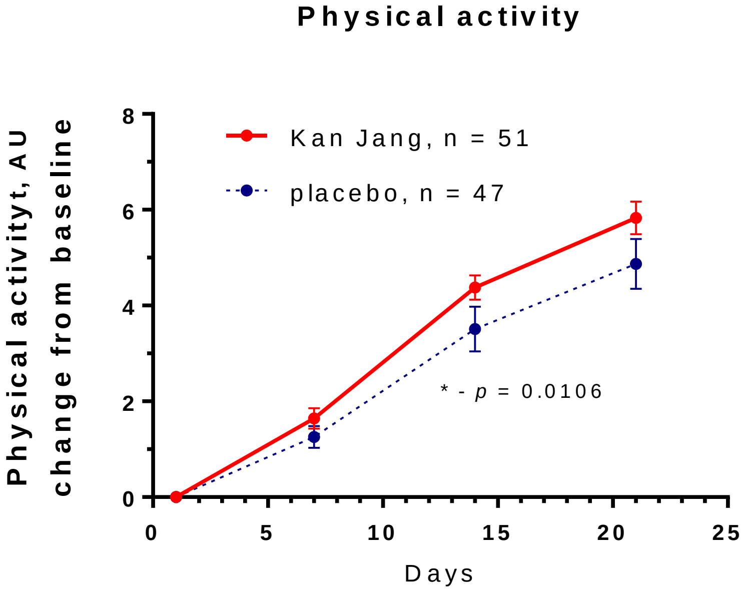

PP analysis in 100 patients

Days	Kan Jang, n = 51			Placebo, n=47		
	Mean	SD	n	Mean	SD	n
1	0	0	53	0	0	47
7	1,642	0,217	53	1,255	0,231	47
14	4,377	0,254	53	3,511	0,467	47
21	5,830	0,343	53	4,872	0,523	47

Workout

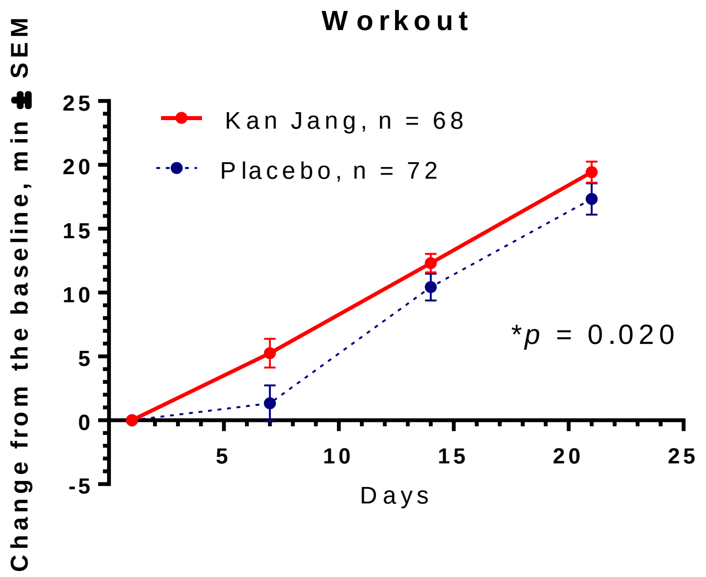

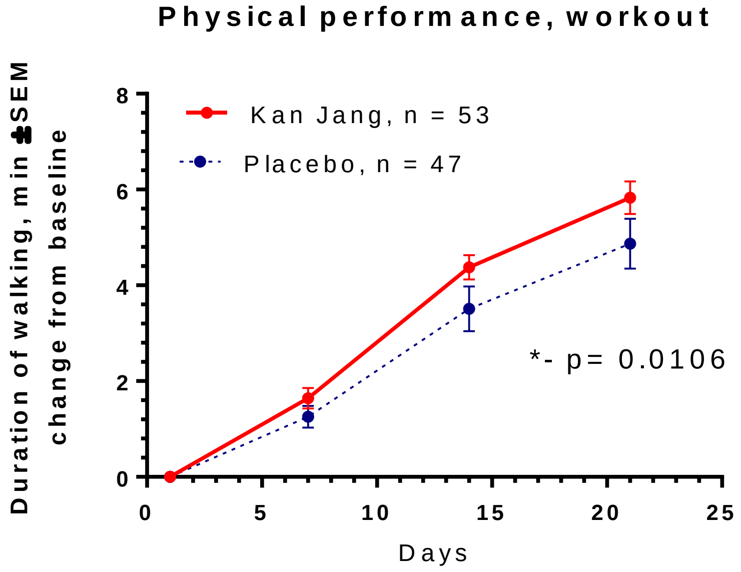

ITT analysis in 140 patients

Days	Kan Jang, n = 68			Placebo, n=72		
	Mean	SD	n	Mean	SD	n
1	0	0	68	0	0	72
7	5,254	1,124	59	1,333	1,411	60
14	12,315	0,733	54	10,426	1,052	47
21	19,434	0,837	53	17,340	1,234	47

PP analysis in 100 patients

Days	Kan Jang, n = 53			Placebo, n=47		
	Mean	SD	n	Mean	SD	n
1	0	0	53	0	0	47
7	1,642	0,217	53	1,255	0,231	47
14	4,377	0,254	53	3,511	0,467	47
21	5,830	0,343	53	4,872	0,523	47

Cognitive performance

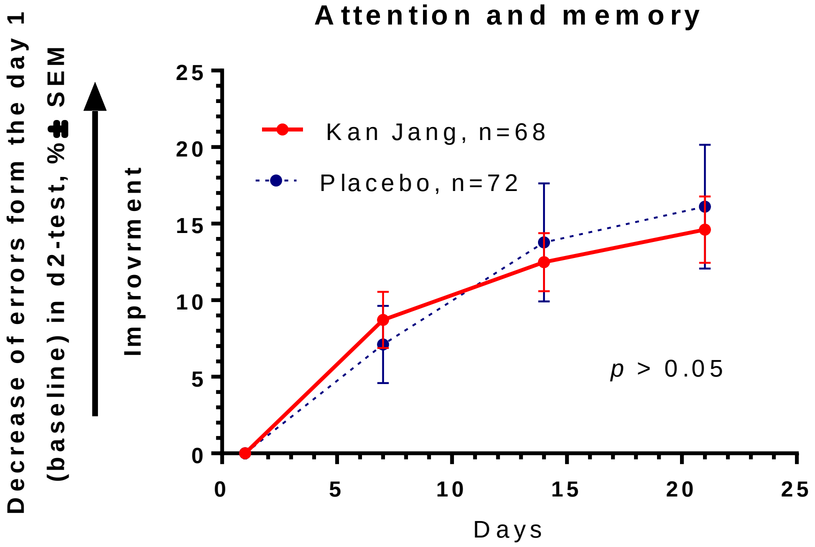

ITT analysis in 140 patients

Days	Kan Jang, n = 27			Placebo, n=15		
	Mean	SD	n	Mean	SD	n
1	0	0	68	0	0	72
7	4,642	3,622	59	5,269	1,481	60
14	8,758	3,201	55	11,227	1,966	47
21	11,305	3,286	54	13,201	2,026	47

PP analysis in 100 patients

Days	Kan Jang, n = 27			Placebo, n=15		
	Mean	SD	n	Mean	SD	n
1	0	0	53	0	0	47
7	1,642	0,217	53	1,255	0,231	47
14	4,377	0,254	53	3,511	0,467	47
21	5,830	0,343	53	4,872	0,523	47

Respiratory symptoms

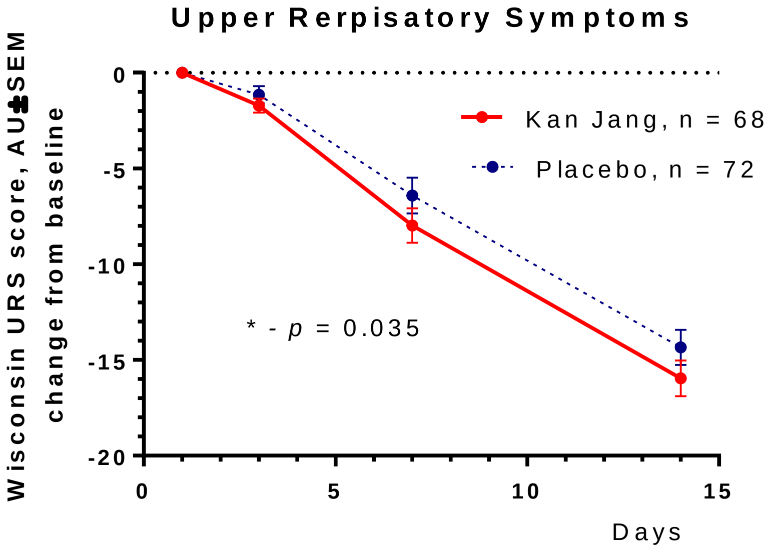

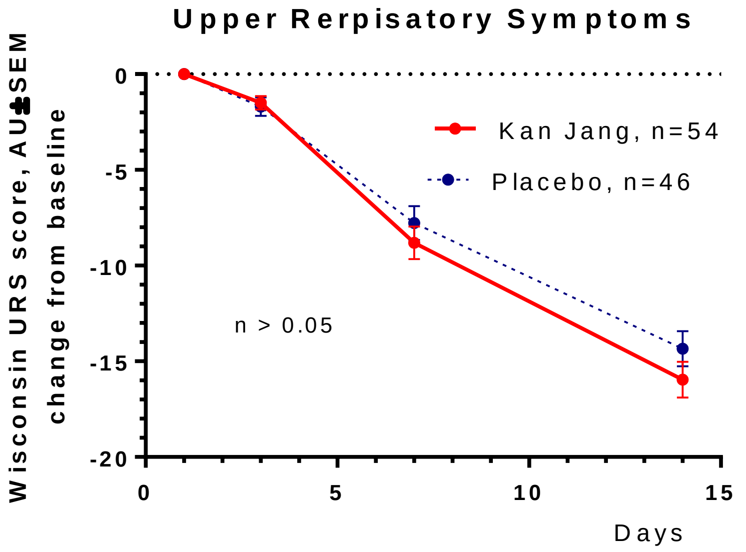

ITT analysis in 140 patients

Days	Kan Jang, n = 27			Placebo, n=15		
	Mean	SD	n	Mean	SD	n
1	0	0	68	0	0	72
3	-1,716	0,369	67	-1,141	0,446	71
7	-7,983	0,900	59	-6,417	0,937	60
14	-15,963	0,936	54	-14,348	0,923	46

PP analysis in 100 patients

Days	Kan Jang, n = 27			Placebo, n=15		
	Mean	SD	n	Mean	SD	n
1	0	0	54	0	0	46
3	-1,500	0,358	54	-1,696	0,480	46
7	-8,815	0,864	54	-7,783	0,890	46
14	-15,963	0,936	54	-14,348	0,923	46

Respiratory symptoms

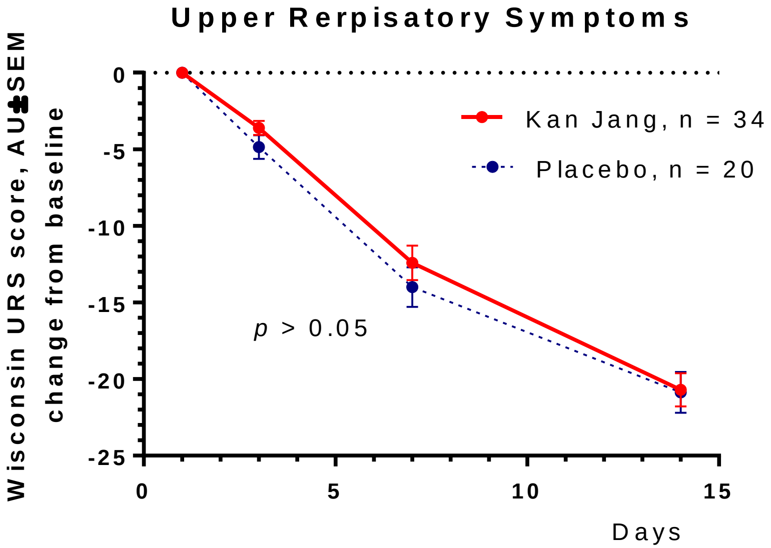

ITT analysis in a subgroup of 54 patients

Days	Kan Jang, n = 27			Placebo, n=15		
	Mean	SD	n	Mean	SD	n
1	0	0	68	0	0	72
7	1,373	0,239	59	0,617	0,341	60
14	4,333	0,254	54	3,511	0,467	47
21	5,830	0,343	53	4,872	0,523	47

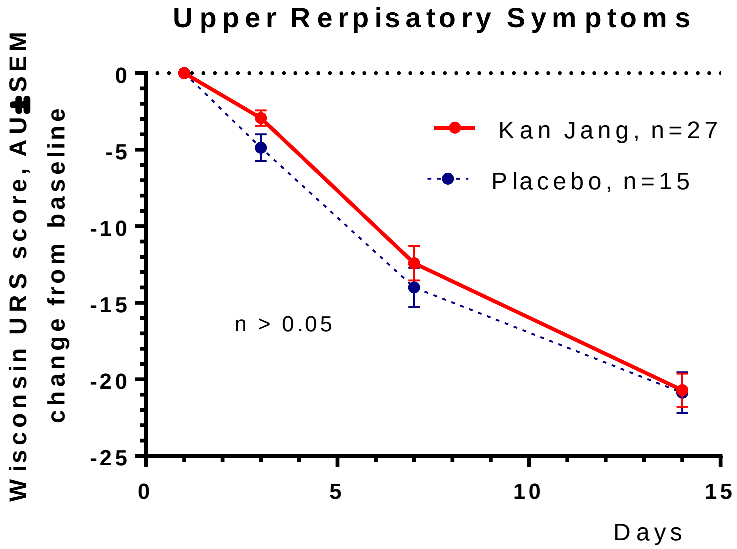

PP analysis in in a subgroup of 42 patients

Days	Kan Jang, n = 27			Placebo, n=15		
	Mean	SD	n	Mean	SD	n
1	0	0	53	0	0	47
7	1,642	0,217	53	1,255	0,231	47
14	4,377	0,254	53	3,511	0,467	47
21	5,830	0,343	53	4,872	0,523	47

Quality of life

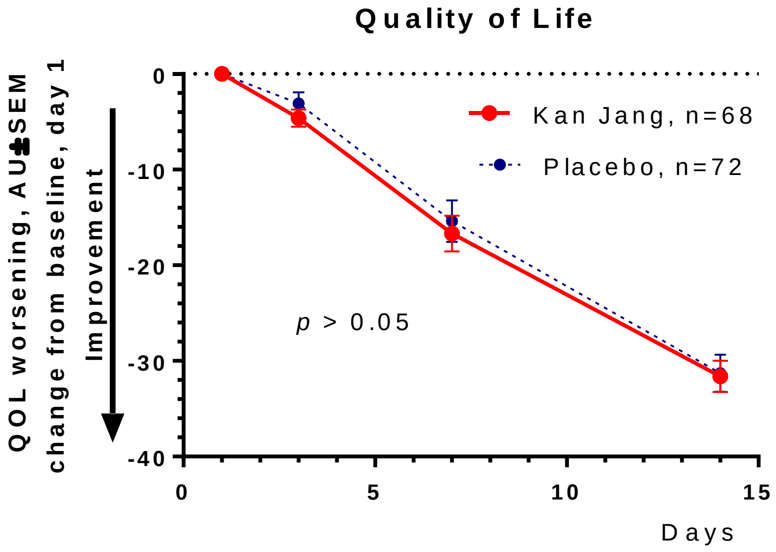

ITT analysis in 140 patients

Days	Kan Jang, n = 68			Placebo, n=72		
	Mean	SD	n	Mean	SD	n
1	0	0	68	0	0	72
7	1,373	0,239	59	0,617	0,341	60
14	4,333	0,254	54	3,511	0,467	47
21	5,830	0,343	53	4,872	0,523	47

PP analysis in 100 patients

Days	Kan Jang, n = 27			Placebo, n=15		
	Mean	SD	n	Mean	SD	n
1	0	0	53	0	0	47
7	1,642	0,217	53	1,255	0,231	47
14	4,377	0,254	53	3,511	0,467	47
21	5,830	0,343	53	4,872	0,523	47

Inflammatory marker IL-6

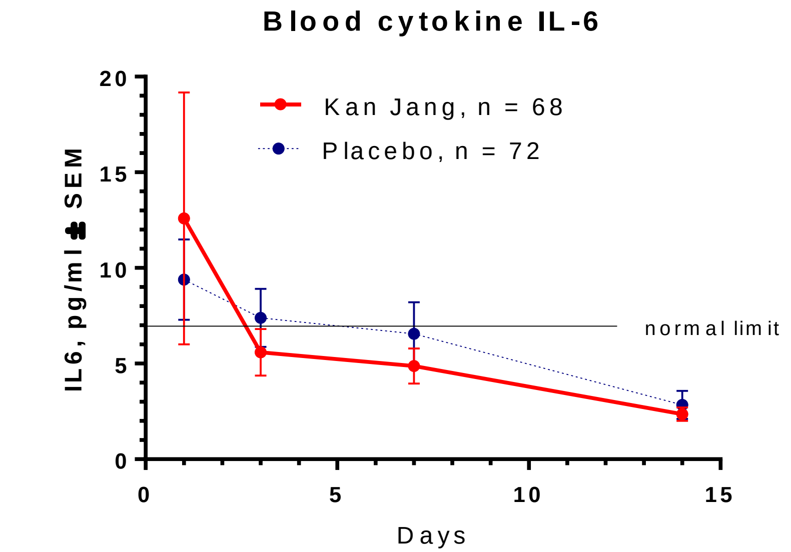

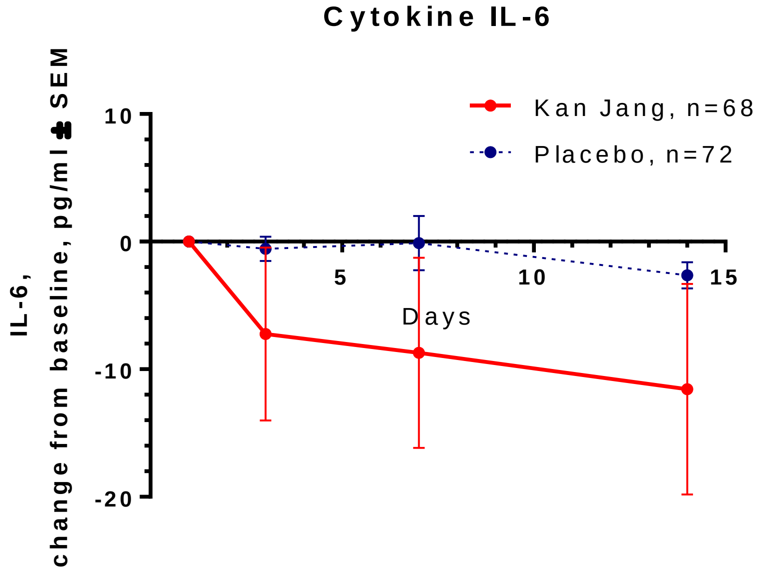

ITT analysis in 140 patients

Days	Kan Jang, n = 68			Placebo, n=72		
	Mean	SD	n	Mean	SD	n
1	12,60	54,29	68	9,39	17,86	72
3	5,59	9,87	66	7,39	12,83	71
7	4,88	7,09	59	6,57	12,77	60
14	2,37	2,60	54	2,84	5,01	47

ITT analysis in 140 patients

Days	Kan Jang, n = 27			Placebo, n=15		
	Mean	SD	n	Mean	SD	n
1	0	0	68	0	0	72
3	-7,235	6,788	66	-0,554	0,970	70
7	-8,702	7,471	59	-0,103	2,131	60
14	-11,575	8,257	54	-2,646	1,037	47

Inflammatory marker IL-6

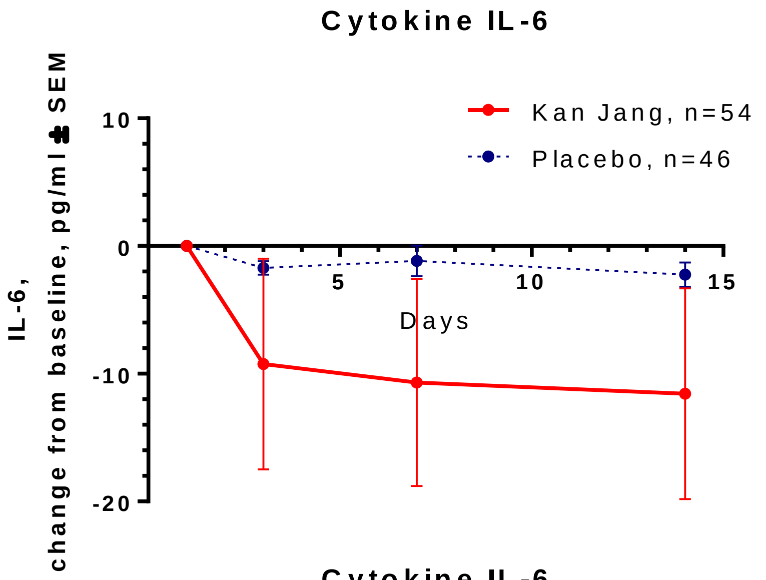

PP analysis in 100 patients

Days	Kan Jang, n = 54			Placebo, n=46		
	Mean	SD	n	Mean	SD	n
1	0	0	54	0	0	46
3	-9,238	8,243	54	-1,712	0,536	46
7	-10,677	8,101	54	-1,168	1,206	46
14	-11,575	8,257	54	-2,229	0,970	46

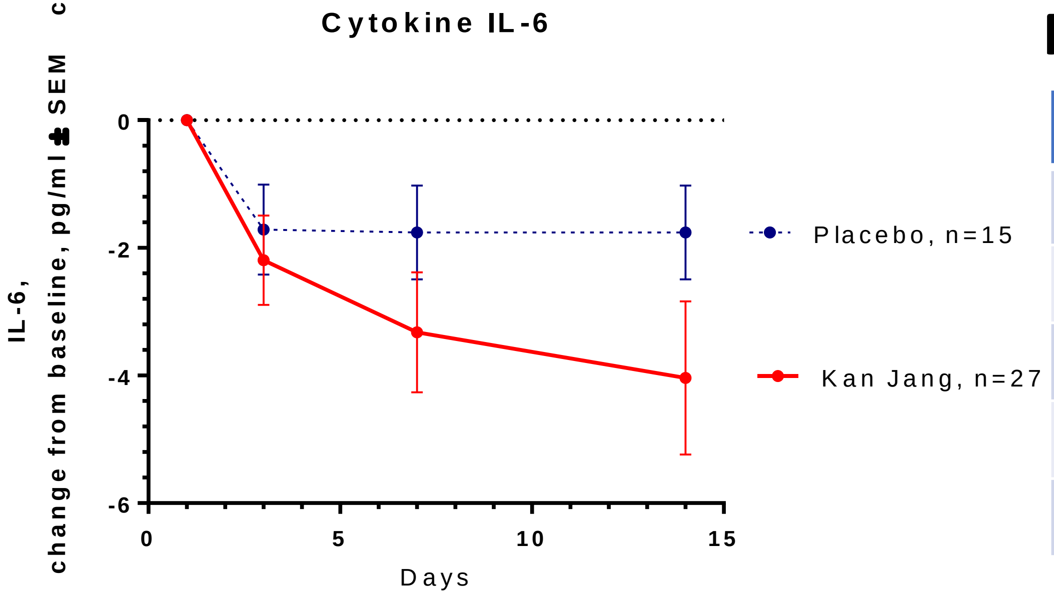

PP analysis in a subgroup of 42 patients

Days	Kan Jang, n = 27			Placebo, n=15		
	Mean	SD	n	Mean	SD	n
1	0	0	27	0	0	15
3	-2,194	0,701	27	-1,711	0,707	15
7	-3,323	0,939	27	-1,758	0,736	15
14	-4,038	1,201	27	-1,758	0,736	15

Inflammatory marker D-dimer

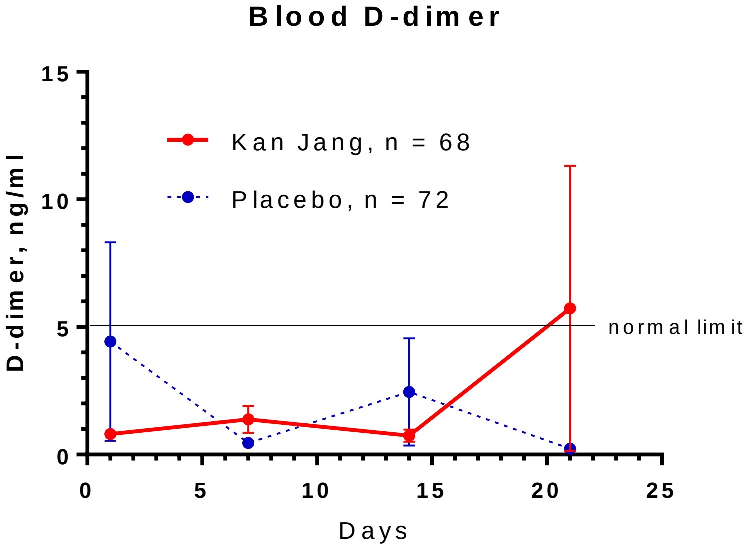

ITT analysis in 140 patients

Days	Kan Jang, n = 27			Placebo, n=15		
	Mean	SD	n	Mean	SD	n
1	0	0	68	0	0	72
7	1,373	0,239	59	0,617	0,341	60
14	4,333	0,254	54	3,511	0,467	47
21	5,830	0,343	53	4,872	0,523	47

PP analysis in 59 patients

Days	Kan Jang, n = 27			Placebo, n=15		
	Mean	SD	n	Mean	SD	n
1	0	0	53	0	0	47
7	1,642	0,217	53	1,255	0,231	47
14	4,377	0,254	53	3,511	0,467	47
21	5,830	0,343	53	4,872	0,523	47

Inflammatory marker C-reactive protein

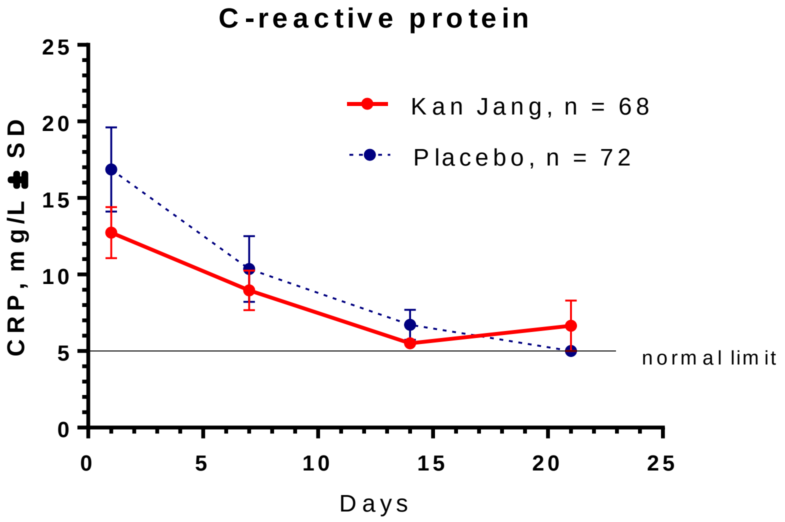

ITT analysis in 140 patients

Days	Kan Jang, n = 27			Placebo, n=15		
	Mean	SD	n	Mean	SD	n
1	0	0	68	0	0	72
7	1,373	0,239	59	0,617	0,341	60
14	4,333	0,254	54	3,511	0,467	47
21	5,830	0,343	53	4,872	0,523	47

PP analysis in 100 patients

Days	Kan Jang, n = 27			Placebo, n=15		
	Mean	SD	n	Mean	SD	n
1	0	0	53	0	0	47
7	1,642	0,217	53	1,255	0,231	47
14	4,377	0,254	53	3,511	0,467	47
21	5,830	0,343	53	4,872	0,523	47

Body temperature

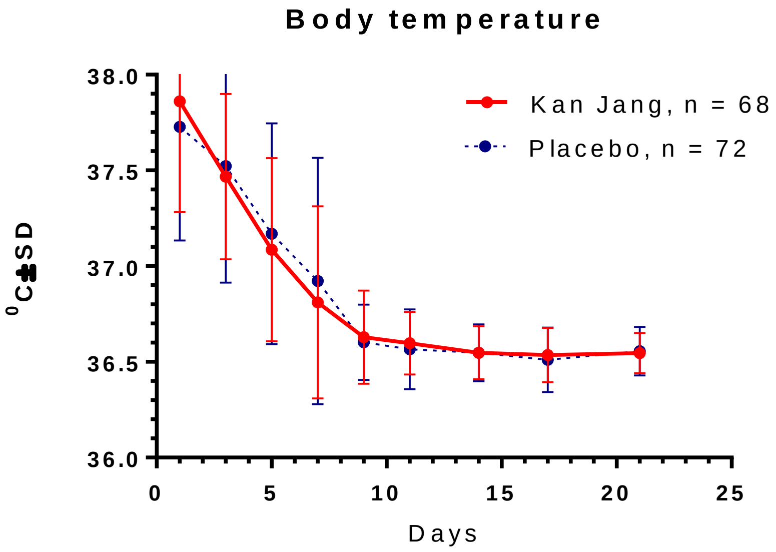

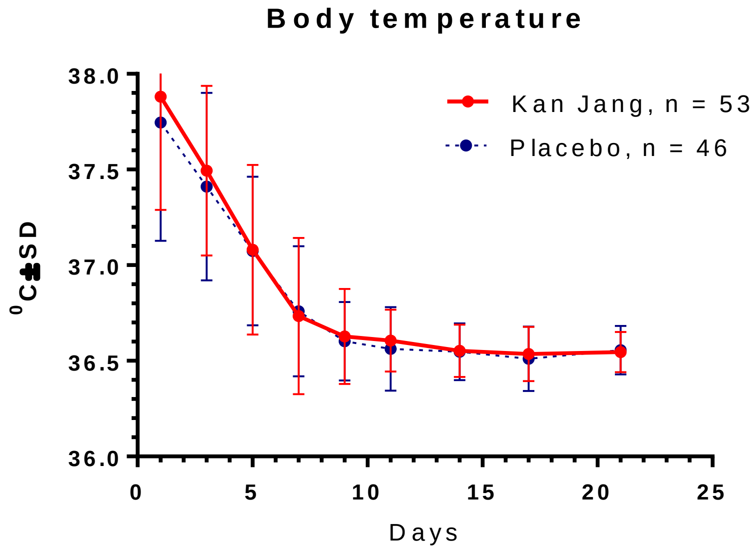

ITT analysis in 140 patients

Days	Kan Jang, n = 27			Placebo, n=15		
	Mean	SD	n	Mean	SD	n
1	0	0	68	0	0	72
7	1,373	0,239	59	0,617	0,341	60
14	4,333	0,254	54	3,511	0,467	47
21	5,830	0,343	53	4,872	0,523	47

PP analysis in 99 patients

Days	Kan Jang, n = 27			Placebo, n=15		
	Mean	SD	n	Mean	SD	n
1	0	0	53	0	0	47
7	1,642	0,217	53	1,255	0,231	47
14	4,377	0,254	53	3,511	0,467	47
21	5,830	0,343	53	4,872	0,523	47

Body temperature

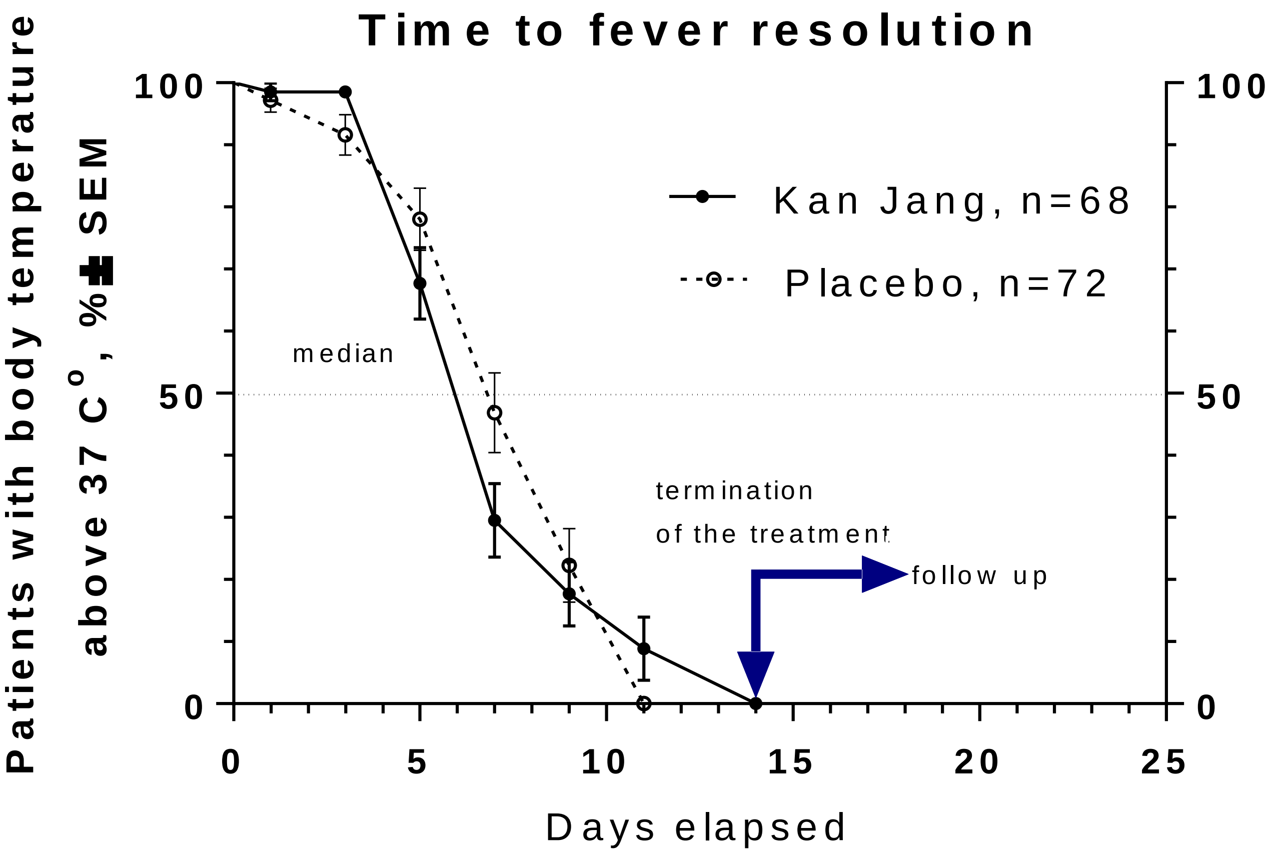

Time to fever resolution				
Days elapsed	Kan Jang, n=68		Placebo, n=72	
0	100		100	
1	98,529	1,460	97,222	1,937
3	98,529		91,586	3,288
5	67,739	5,796	78,073	5,015
7	29,527	5,943	46,844	6,447
9	17,716	5,164	22,307	5,957
11	8,858	5,127	0,000	0,000
14	0	0		

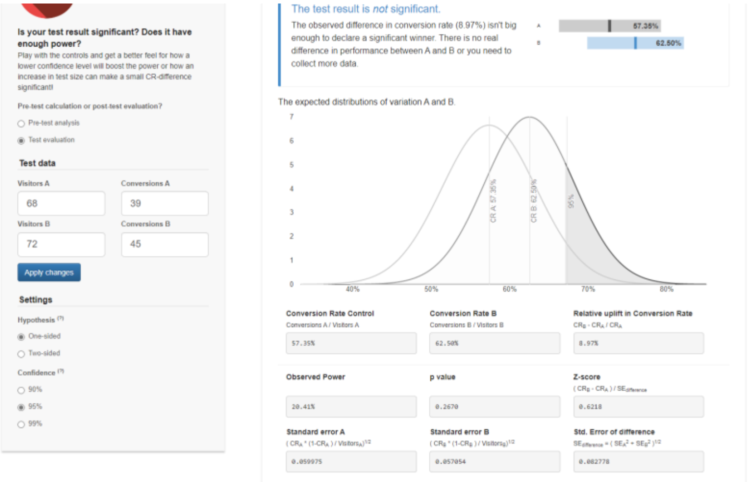

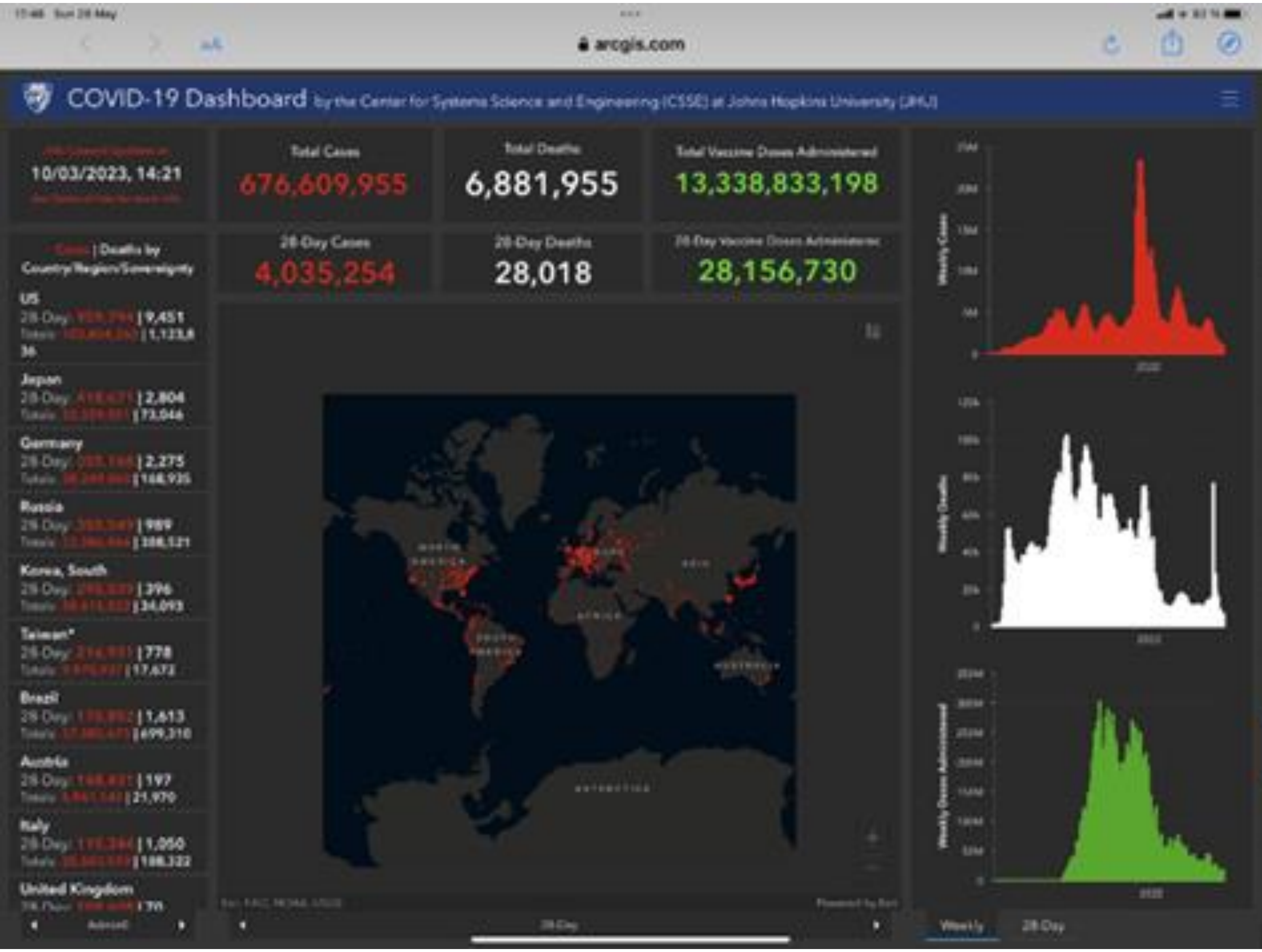
arcgis.com
COVID-19 Dashboard by the Center for Systems Science and Engineering (CSSE) at Johns Hopkins University (JHU)
10/03/2023, 14:21
Total Cases
676,609,955
Total Deaths
6,881,955
Total Vaccine Doses Administered
13,338,833,198
28-Day Cases
4,035,254
28-Day Deaths
28,018
28-Day Vaccine Doses Administered
28,156,730
US
Japan
Germany
Russia
Korea, South
Taiwan*
Brazil
Austria
Italy
United Kingdom
Weekly
28-Day
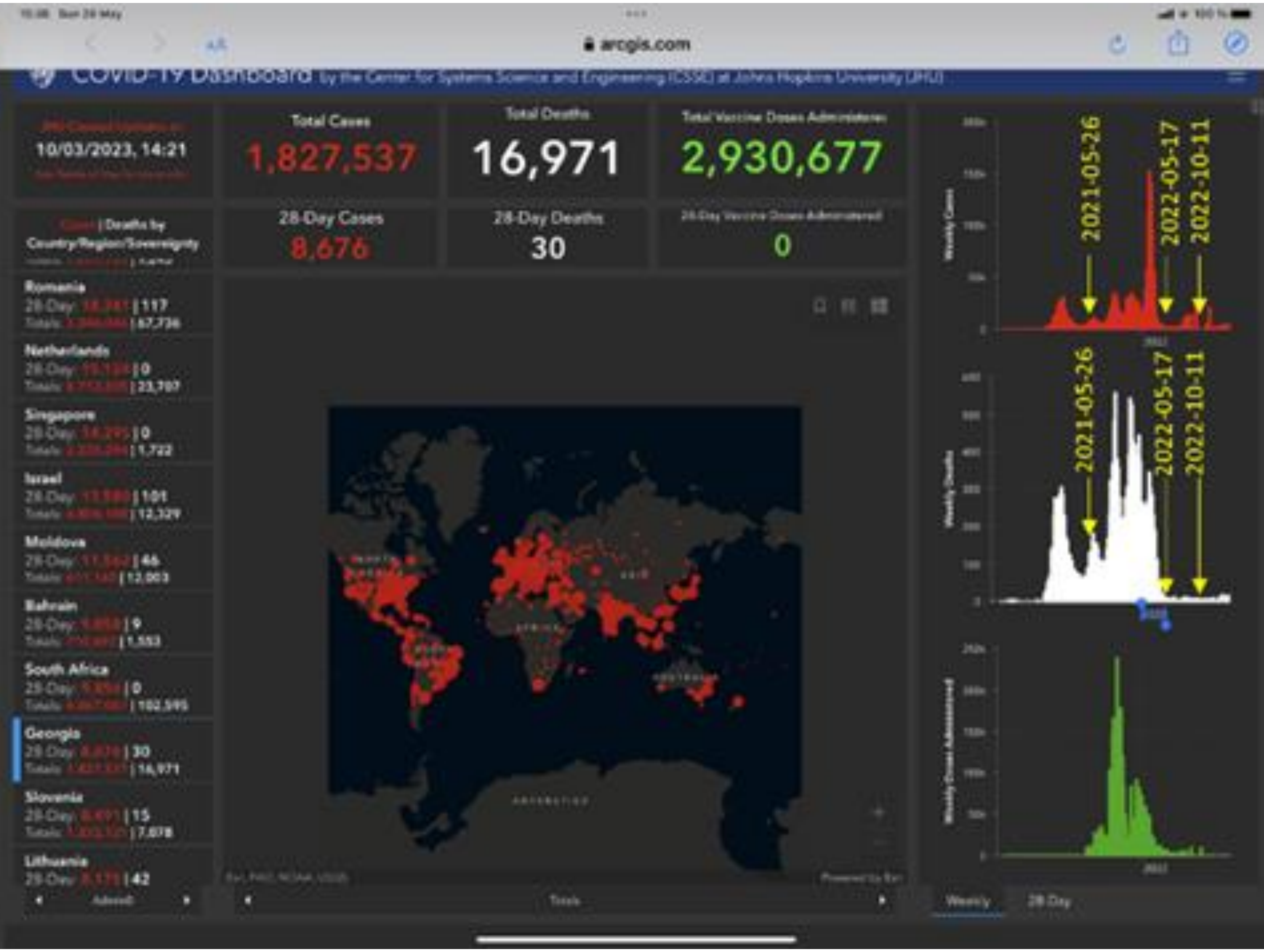
arcgis.com
10/03/2023, 14:21
Total Cases
1,827,537
Total Deaths
16,971
Total Vaccine Doses Administered
2,930,677
28-Day Cases
8,676
28-Day Deaths
30
0
Romania
Netherlands
Singapore
Israel
Moldova
Bahrain
South Africa
Georgia
Slovenia
Lithuania
2021-05-26
2022-05-17
2022-10-11
2021-05-26
2022-05-17
2022-10-11
Weekly
28-Day

Conditional “biological signatures” of Andrographis, Eleutherococcus and their combination Kan Jang

The color-coded heat-maps identify diseases and functions that are expected to increase or decrease.

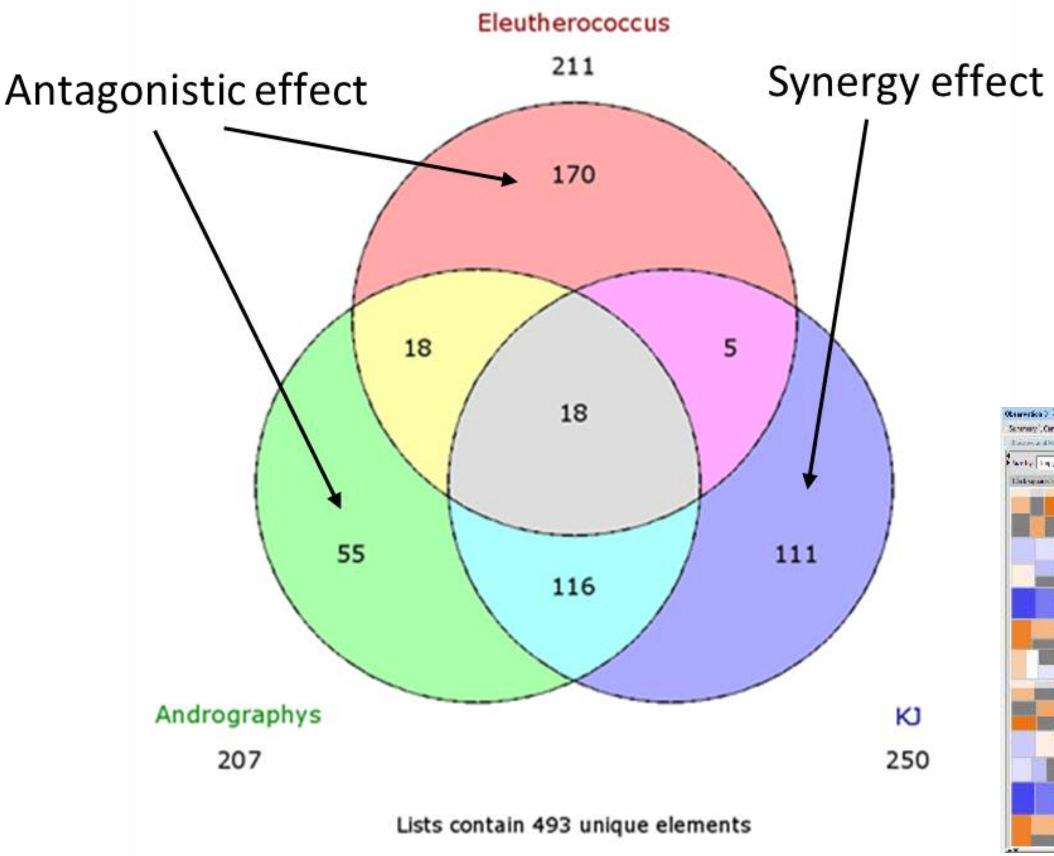

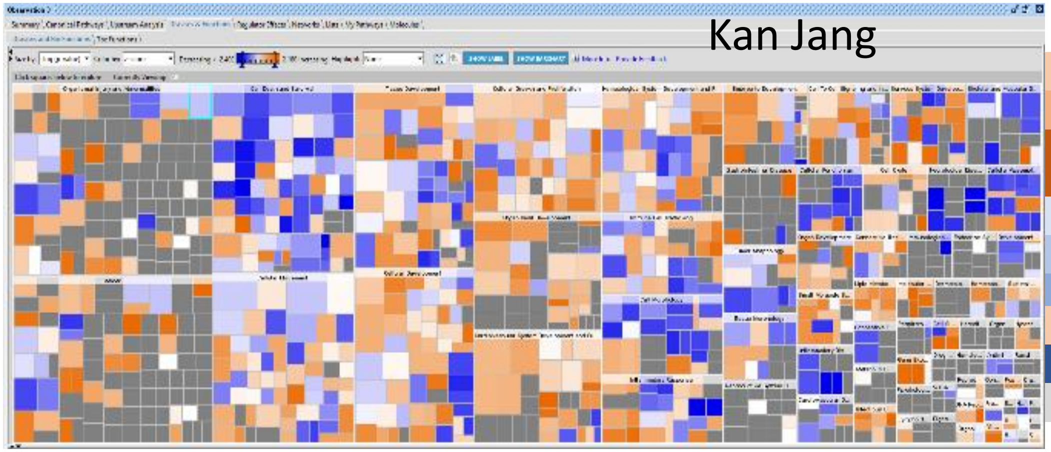

Diseases or Functions

- antigen presentation
- clearance of virus
- antiviral response of myeloid cells
- emotional behavior
- locomotion
- active avoidance response
- sexual receptivity
- aggressive behavior
- appetite, etc.

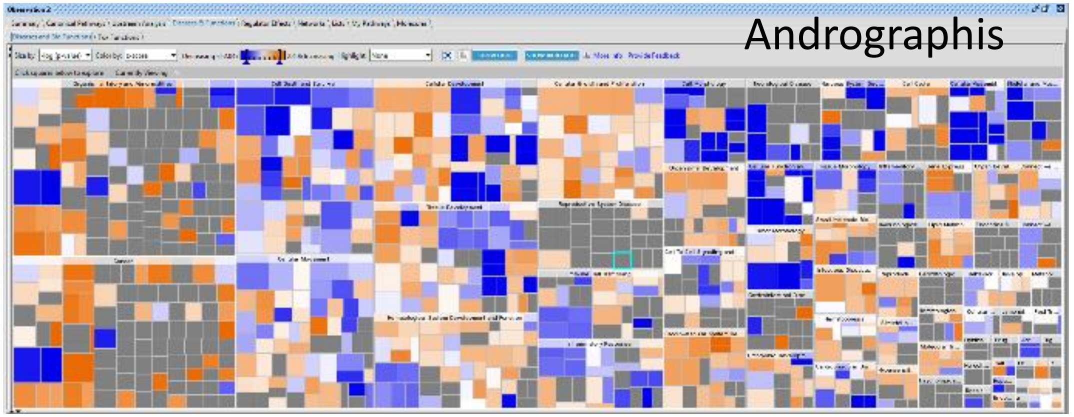

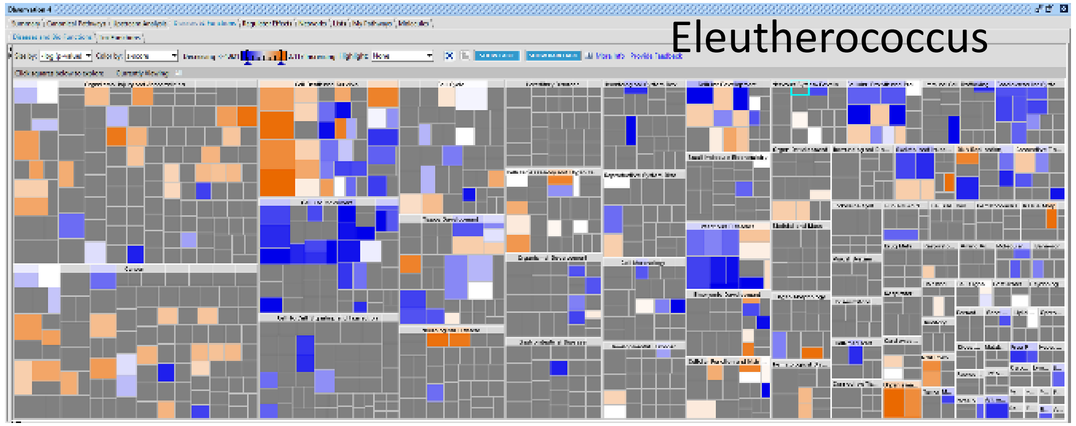

Direct antiviral effect

- Effects on viral life cycle in infected host cells – targets preventing the virus RNA synthesis and replication
- inhibiting virus structural proteins
- Viricidal effects

Anti-inflammatory activity

- Inhibition of NFkB mediated signaling
- Inhibition of PLA2, arachidonic acid release and metabolism
- Inhibition of nitric oxide generation

Andrographis Eleutherococcus

Modulation of the immune response

- Increased expression of defensins peptides,
- Increased expression of pathogen pattern recognition proteins - TLRs,
- Increased expression of interferons,
- Inhibition of IL-6, IL-1β, IL-10, TNF release,
- Inhibition of NFkB
- Activation natural killer cells,
- Activation of phagocyting cells,
- Activation of T- and B - lymphocytes
- Activation of melatonin signaling

Detoxification and reparation of oxidative stress induced damages in compromised cells

- Activation of NRF2-signaling pathway proteins (KEAP1)
- Production of Phases I,II metabolizing and antioxidant enzymes : glutathione S-transferase (GST), NAD(P)H quinone oxidoreductase 1 (NQO1) , superoxide dismutase (SOD), and heme oxygenase 1 (HO1).
- Molecular chaperons Hsp70 mediated cytoprotecting, and repairing processes
- Activation of melatonin signaling pathway

Eleutherococcus senticosus
(Rupr. and Maxim.) Maxim.

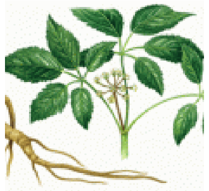

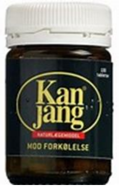

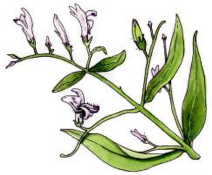

Andrographis paniculata
(Burm. f.) Nees

Placebo.

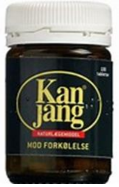

Randomization
masking/blinding

140 patients
randomly allocated
to Kan Jang or
placebo

100 patients
Completed study

The overall clinical effectiveness:
Kan Jang : Placebo = 3.84

Number patients
needed to treat = 8

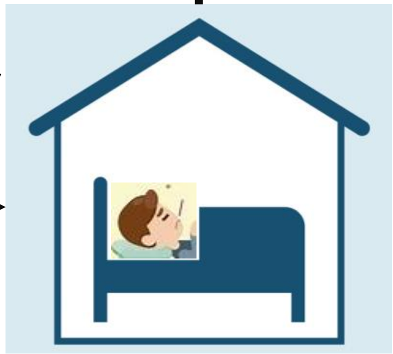

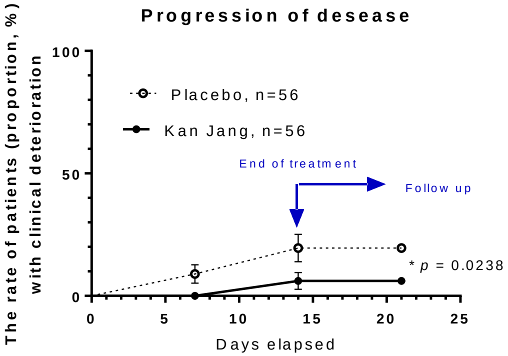

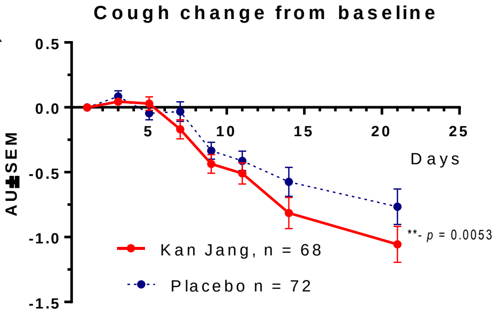

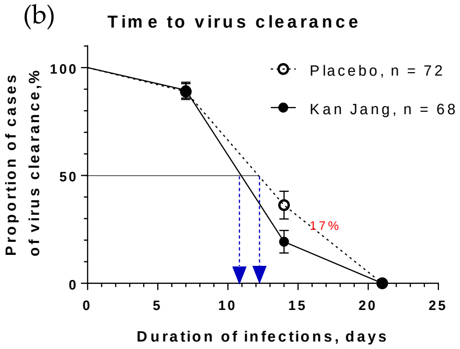

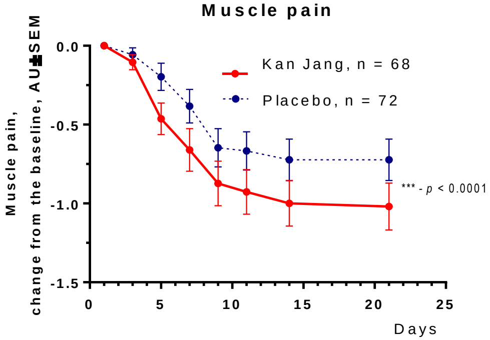

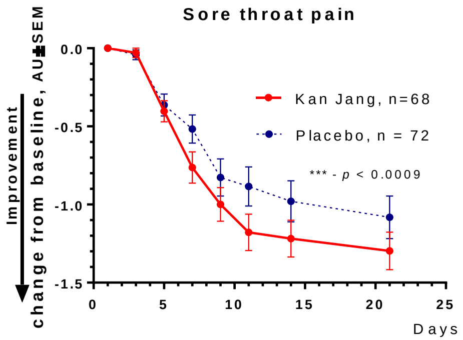

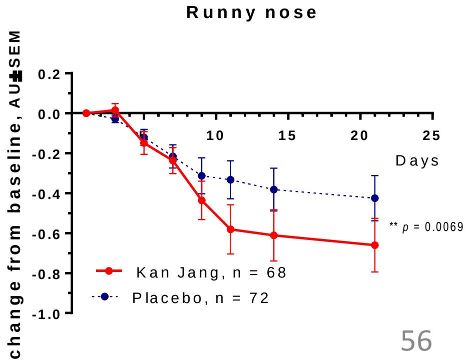

Summary of the results

Kan Jang significantly ameliorates the overall recovery rate by decreasing the following:

- the rate of cases turning to severe,
- negative conversion rate of SARS CoV-2 virus detection.

That is accompanied with decreasing the duration of sick days at the clinic or home compared with the placebo group.

Disappearance rate of inflammatory symptom in the Kan Jang group was significantly improved compared with the placebo group, including:

Beneficial effect of Kan Jang

- Severity of Respiratory symptoms - Wisconsin URS score
- Severity of Cough
- Severity of Sore throat pain
- Severity of Runny nose
- Severity of Muscle soreness
- Loss of smell
- Physical activity
- Workout
- IL6 - only in the subgroup of 86 patients with moderate symptoms, but the effect was statistically insignificant in the sample size of 140 patients with mild to moderate symptoms of COVID-19

No effect on

- cognitive functions (attention and memory)
- quality of life score
- inflammatory markers D-dimer and c reactive protein
- time to normalization of body temperature
- severity of Fatigue
- severity of Headache
