# Supplementary material for: Efficacy of Kan Jang® in Patients with Mild COVID-19: A Randomized, Quadruple-Blind, Placebo-Controlled Trial
Source: Pharmaceuticals (Basel). 2023 Aug 22;16(9):1196. doi: 10.3390/ph16091196 (PMC10535596; doi:10.3390/ph16091196)
Supplement: Supplementary file 1 [file pharmaceuticals-16-01196-s001.zip › Supplement 3 Safety.pdf]

SAFETY EVALUATION

Extent of Exposure

Duration: 1-14 days

Daily Dose: 6 capsules daily (two capsules three times per day for two consecutive weeks), in total of 1672,5 mg of fixed combination of 1560 mg proprietary *Andrographis paniculata* L. Nees. herb standardized native extract (corresponding to 90 mg of diterpene lactones andrographolides), and 112.5 mg proprietary *Eleutherococcus senticosus* root native extract, equivalent to 7-12.6 g of Herba Andrographidis, and 1.9-3.4 g of Radix Eleutherococci.

2 Adverse Events (AEs)

Summary of adverse events

The treatment was well tolerated. No side effects were observed in 140 patients during the treatment in both study groups. Serious adverse events were not observed.

Display of adverse events

No adverse events of allergic reactions like urticaria, angioedema, paresthesia, anaphylactic reactions, rush, and pruritus (itchy skin), were observed after initiation of study treatments, including events likely to be related to the underlying disease or likely to represent concomitant illness are displayed in summary tables 1 - 3.

Deaths, Other Serious Adverse Events, and Other Significant Adverse Events

Deaths, other serious adverse events, and other significant adverse deserve special attention have not been observed in this study.

Clinical Laboratory Evaluation

The results of blood analysis shows normalizing (anti-inflammatory) effect of Kan Jang.

Vital Signs, Physical Findings, and Other Observations Related to Safety

Vital signs related to safety do not reveal any evidence of a drug effect.

Safety Conclusions

The treatment was well tolerated. No side effect was observed in 140 patients during the treatment in both study groups. Serious adverse events were not observed.

Table 1. Adverse events: treatment emergent signs and symptoms" (TESS) - those not seen at baseline. Number observed and rate with patient identification. Kan Jang group, N=68

Adverse event	Mild		Moderate		Severe		Total		Total
	Possibly Related	NR*	Possibly Related	NR	Possibly Related	NR	Possibly Related	NR	R+NR *
none									

** NR = not related; R – possibly related ** Patient identification number*

Table 2. Adverse events: treatment emergent signs and symptoms" (TESS) - those not seen at baseline. Number observed and rate with patient identification. Placebo group, N=72

Adverse event	Mild		Moderate		Severe		Total		Total
	Possibly Related	NR*	Possibly Related	NR	Possibly Related	NR	Possibly Related	NR	R+NR

Table 3. Overall summary of the treatment-emergent adverse events

	Group A (Kan Jang)	Group B (Placebo)	Total	Odds ratio, B/A	z statistic	Significance level, *p* value
Number of patients	68	72	140			
Patients with AE	0 (0%)	0 (0%)	0 (0%)			
Total number of AEs: pruritus/ itchy skin	0 (0%)	0 (0%)	0 (0%)			
Number of patients with other serious AEs	0 (0%)	0 (0%)	0 (0%)			
Number of patients withdrawn for AEs	0 (0%)	0 (0%)	0 (0%)			
Cases of death due to AEs	0 (0%)	0 (0%)	0 (0%)			
