# Supplementary material for: Efficacy of Kan Jang® in Patients with Mild COVID-19: A Randomized, Quadruple-Blind, Placebo-Controlled Trial
Source: Pharmaceuticals (Basel). 2023 Aug 22;16(9):1196. doi: 10.3390/ph16091196 (PMC10535596; doi:10.3390/ph16091196)
Supplement: Supplementary file 1 [file pharmaceuticals-16-01196-s001.zip › Supplement 5 KJ Compliance 140.pdf]

Investigational Product (IP) Compliance

Summary Table*

**Treatment** Random No.	**Participant name**	**IP dispensed, capsules**	**IP returned, capsules**	**IP consumed, capsules**	**Compliance, %**	**Treatment group***	**KJ group A Compliance, %**	**Placebo group B Compliance, %**
1	TI_ MC	90	6	84	**100**	A	100	
2	NI_BA	90	6	84	**100**	A	100	
3	NI_ GO	90	6	84	**100**	A	100	
4	MA_NE	90	6	84	**100**	A	100	
5	NI_DE	90	6	84	**100**	B		100
6	NI_ TA	90	84	6	**100**	A	7	
7	NA_ MI	90	6	84	**100**	B		100
8	NA_AB	90	48	42	**100**	B		50
9	MA_ TI	90	48	42	**100**	B		50
10	NA_GO	90	54	36	**100**	B		43
11	OL_KH	90	48	42	**100**	B		50
12	GO_MA	90	6	84	**100**	B		100
13	NA_ AK	90	6	84	**100**	A	100	
14	GI_GI	90	48	42	**100**	B		50
15	RE_KA	90	48	42	**100**	B		50
16	LE_JI	90	6	84	**100**	B		100
17	TI_ MC	90	6	84	**100**	B		100
18	NI_BA	90	6	84	**100**	B		100
19	NI_ GO	90	6	84	**100**	B		100
20	MA_NE	90	6	84	**100**	A	100	

* - Dropout patients are highlighted on gray background

**Treatment** Random No.	**Participant name**	**IP dispensed, capsules**	**IP returned, capsules**	**IP consumed, capsules**	**Compliance, %**	**Treatment group***	**KJ group A Compliance, %**	**Placebo group B Compliance, %**
21	MA_GU	90	48	42	**100**	A	50	
22	OT_KH	90	6	84	**100**	A	100	
23	MA_DA	90	6	84	**100**	A	100	
24	SA_ BA	90	6	84	**100**	B		100
25	TA_PA	90	6	84	**100**	B		100
26	LE_BI	90	6	84	**100**	B		100
27	NA_BI	90	48	42	**100**	A	50	
28	TA_GZ	90	6	84	**100**	A	100	
29	DA_KA	90	48	42	**100**	A	50	
30	GI_GE	90	6	84	**100**	B		100
31	NU_AB	90	6	84	**100**	B		100
32	MI_AB	90	12	78	**100**	B		93
33	NI_GE	90	6	84	**100**	B		100
34	NI_GV	90	6	84	**100**	B		100
35	EL_KA	90	84	6	**100**	B		7
36	BE_KE	90	6	84	**100**	A	100	
37	IN_GR	90	6	84	**100**	B		100
38	GI_CH	90	6	84	**100**	A	100	
39	LE_TA	90	6	84	**100**	A	100	
40	FI_KH	90	42	48	**100**	A	57	

* - Dropout patients are highlighted on gray background

**Treatment** Random No.	**Participant name**	**IP dispensed, capsules**	**IP returned, capsules**	**IP consumed, capsules**	**Compliance, %**	**Treatment group***	**KJ group A Compliance, %**	**Placebo group B Compliance, %**
41	TA_SH	90	74	16	**100**	B		19
42	SO_TO	90	6	84	**100**	B		100
43	IR_JA	90	6	84	**100**	B		100
44	IA_MA	90	6	84	**100**	B		100
45	KE_RI	90	6	84	**100**	B		100
46	MU_AB	90	6	84	**100**	B		100
47	RO_AB	90	6	84	**100**	A	100	
48	GI_SO	90	6	84	**100**	B		100
49	LI_ZA	90	6	84	**100**	B		100
50	NA_RO	90	6	84	**100**	B		100
51	NI_BR	90	62	18	**112.5**	B		21
52	IA_KU	90	62	18	**112.5**	B		21
53	MZ_SI	90	62	18	**112.5**	B		21
54	AM_KE	90	62	18	**112.5**	B		21
55	ZA_KU	90	6	84	**100**	B		100
56	DE_CH	90	6	84	**100**	A	100	
57	DA_KV	90	6	84	**100**	B		100
58	NI_NI	90	6	84	**100**	A	100	
59	IL_ME	90	6	84	**100**	A	100	
60	DA_GO	90	6	84	**100**	B		100

* - Dropout patients are highlighted on gray background

**Treatment** Random No.	**Participant name**	**IP dispensed, capsules**	**IP returned, capsules**	**IP consumed, capsules**	**Compliance, %**	**Treatment group***	**KJ group A Compliance, %**	**Placebo group B Compliance, %**
61	TA_RE	90	6	84	**100**	A	100	
62	EK_GE	90	6	84	**100**	B		100
63	BE_VA	90	6	84	**100**	A	100	
64	MA_CH	90	6	84	**100**	B		100
65	GI_UG	90	6	84	**100**	B		100
66	IN_SU	90	6	84	**100**	B		100
67	TA_KA	90	6	84	**100**	B		100
68	TE_MO	90	6	84	**100**	B		100
69	TE_SI	90	6	84	**100**	A	100	
70	TA_BA	90	6	84	**100**	B		100
71	DE_SI	90	48	42	**100**	B		50
72	NA_NA	90	48	42	**100**	B		50
73	MA_KA	90	6	84	**100**	A	100	
74	NI_BA	90	6	84	**100**	A	100	
75	TE_GE	90	42	48	**100**	B		57
76	NA_KA	90	6	84	**100**	A	100	
77	TA_BU	90	6	84	**100**	A	100	
78	NA_ZA	90	6	84	**100**	A	100	
79	AN_AL	90	54	36	**100**	A	43	
80	MA_AS	90	6	84	**100**	A	100	

* - Dropout patients are highlighted on gray background

**Treatment** Random No.	**Participant name**	**IP dispensed, capsules**	**IP returned, capsules**	**IP consumed, capsules**	**Compliance, %**	**Treatment group***	**KJ group A Compliance, %**	**Placebo group B Compliance, %**
81	TI_LO	90	6	84	**100**	B		100
82	LI_LO	90	6	84	**100**	B		100
83	LI_KA	90	6	84	**100**	A	100	
84	SH_VA	90	6	84	**100**	A	100	
85	NI_VA	90	6	84	**100**	B		100
86	SH_PO	90	6	84	**100**	A	100	
87	NI_MO	90	6	84	**100**	A	100	
88	TE_UK	90	6	84	**100**	B		100
89	NI_CH	90	6	84	**100**	A	100	
90	GI_CH	90	6	84	**100**	A	100	
91	TA_KA	90	6	84	**100**	A	100	
92	AN_VA	90	6	84	**100**	B		100
93	AN_KH	90	6	84	**100**	A	100	
94	SH_KA	90	6	84	**100**	A	100	
95	LA_CH	90	6	84	**100**	A	100	
96	TA_JO	90	6	84	**100**	B		100
97	MA_TE	90	6	84	**100**	A	100	
98	GI_SO	90	6	84	**100**	A	100	
99	IZ_LA	90	6	84	**100**	A	100	
100	GR_GU	90	6	84	**100**	A	100	

* - Dropout patients are highlighted on gray background

**Treatment** Random No.	**Participant name**	**IP dispensed, capsules**	**IP returned, capsules**	**IP consumed, capsules**	**Compliance, %**	**Treatment group***	**KJ group A Compliance, %**	**Placebo group B Compliance, %**
101	NI_OT	90	6	84	**100**	A	100	
102	SH_MU	90	6	84	**100**	A	100	
103	MA_KU	90	6	84	**100**	B		100
104	GI_KA	90	6	84	**100**	A	100	
105	KE_MO	90	6	84	**100**	B		100
106	SA_OR	90	54	36	**100**	B		43
107	LE_OR	90	54	36	**100**	B		43
108	NA_OR	90	54	36	**100**	B		43
109	TA_ KU	90	6	84	**100**	B		100
110	NU_GU	90	6	84	**100**	B		100
111	NA_GU	90	6	84	**100**	A	100	
112	NA_ME	90	6	84	**100**	B		100
113	TO_KE	90	6	84	**100**	B		100
114	AL_NA	90	6	84	**100**	A	100	
115	NI_NA	90	6	84	**100**	B		100
116	TI_BA	90	6	84	**100**	A	100	
117	MZ_KA	90	6	84	**100**	A	100	
118	VL_BA	90	6	84	**100**	B		100
119	GI_KA	90	6	84	**100**	B		100
120	TI_NE	90	54	36	**100**	B		43

* - Dropout patients are highlighted on gray background

**Treatment** Random No.	**Participant name**	**IP dispensed, capsules**	**IP returned, capsules**	**IP consumed, capsules**	**Compliance, %**	**Treatment group***	**KJ group A Compliance, %**	**Placebo group B Compliance, %**
121	NE_SA	90	54	36	**100**	A	43	
122	KR_SA	90	54	36	**100**	B		43
123	TA_GE	90	6	84	**100**	A	100	
124	MA_GE	90	54	36	**100**	A	43	
125	NA_LA	90	54	36	**100**	A	43	
126	TA_KH	90	6	84	**100**	B		100
127	NI_KA	90	6	84	**100**	A	100	
128	NA_GA	90	54	36	**100**	A	43	
129	EK_GE	90	6	84	**100**	B		100
130	MA_AR	90	6	84	**100**	A	100	
131	LE_BA	90	6	84	**100**	B		100
132	MA_KV	90	6	84	**100**	A	100	
133	KN_TO	90	6	84	**100**	A	100	
134	IO_GA	90	6	84	**100**	A	100	
135	KH_KA	90	6	84	**100**	A	100	
136	LA_NA	90	6	84	**100**	A	100	
137	TA_TU	90	54	36	**100**	A	43	
138	DI_ON	90	54	36	**100**	A	43	
139	EK_ON	90	54	36	**100**	A	43	
140	EK_DE	90	6	84	**100**	A	100	
**ITT analysis: medication compliance,** %, mean ± SD, n – 140 patients							**89.0±2.8**	**82.0±3.4**
Significance of difference Kan Jang *vs* placebo, *p* value							**0.2887^{ns}**	
**IPP analysis: medication compliance,** %, mean ± SD, n – 100 patients							**100**	**100**
Significance of difference Kan Jang vs placebo, *p* value							**ns**	

Principal Investigator ______ ______ **Study Monitor** ______ ______

(Date, Signature) Prof. Levan Ratiani, PhD, MD Prof. Areg Hovhannisyan
