# Supplementary material for: Efficacy of Kan Jang® in Patients with Mild COVID-19: A Randomized, Quadruple-Blind, Placebo-Controlled Trial
Source: Pharmaceuticals (Basel). 2023 Aug 22;16(9):1196. doi: 10.3390/ph16091196 (PMC10535596; doi:10.3390/ph16091196)
Supplement: Supplementary file 1 [file pharmaceuticals-16-01196-s001.zip › Supplement 4 CONSORT-checklist.pdf]

CONSORT 2010 checklist

Section/Topic	Item No	Checklist item	Reported on page No
**Title and abstract**			
	1a	Identification as a randomized trial in the title	1
	1b	Structured summary of trial design, methods, results, and conclusions (for specific guidance, see CONSORT for abstracts)	1
**Introduction**			
Background and objectives	2a	Scientific background and explanation of the rationale	2,3 Figure 1
	2b	Specific objectives or hypotheses	3,4 Figures 2,3
**Methods**			
Trial design	3a	Description of trial design (such as parallel, factorial), including allocation ratio	14, Figure 4
	3b	Important changes to methods after trial commencement (such as eligibility criteria), with reasons	n/a
Participants	4a	Eligibility criteria for participants	15,16
	4b	Settings and locations where the data were collected	14
Interventions	5	The interventions for each group with sufficient details to allow replication, including how and when they were actually administered	14
Outcomes	6a	Completely defined pre-specified primary and secondary outcome measures, including how and when they were assessed	17,18, Table 2
	6b	Any changes to trial outcomes after the trial commenced, with reasons	n/a
Sample size	7a	How sample size was determined	14, Ref 18
	7b	When applicable, explanation of any interim analyses and stopping guidelines	Ref 18
Randomization:			
Sequence generation	8a	The method used to generate the random allocation sequence	16,17
	8b	Type of randomization; details of any restriction (such as blocking and block size)	17
Allocation concealment mechanism	9	The mechanism used to implement the random allocation sequence (such as sequentially numbered containers), describing any steps taken to conceal the sequence until interventions were assigned	17
Implementation	10	Who generated the random allocation sequence, who enrolled participants, and who assigned participants to interventions?	17
Blinding	11a	If done, who was blinded after assignment to interventions (for example, participants, care providers, those	17

		assessing outcomes), and how	
	11b	If relevant, a description of the similarity of interventions	17
Statistical methods	12a	Statistical methods used to compare groups for primary and secondary outcomes	14, Ref 18
	12b	Methods for additional analyses, such as subgroup analyses and adjusted analyses	14, Ref 18
**Results**			
Participant flow (a diagram is strongly recommended)	13a	For each group, the number of participants who were randomly assigned received the intended treatment and was analyzed for the primary outcome.	4, Figure 4, Table 1
	13b	For each group, losses and exclusions after randomization, together with reasons	4, Figure 4
Recruitment	14a	Dates defining the periods of recruitment and follow-up	4,14, Figure 3
	14b	Why the trial ended or was stopped	4,14, Figure 3
Baseline data	15	A table showing baseline demographic and clinical characteristics for each group	Table 1
Numbers analyzed	16	For each group, the number of participants (denominator) included in each analysis and whether the analysis was by originally assigned groups	Figures 5-13
Outcomes and estimation	17a	For each primary and secondary outcome, results for each group, and the estimated effect size and its precision (such as 95% confidence interval)	Figures 5-13 pages 6-12
	17b	For binary outcomes, the presentation of both absolute and relative effect sizes is recommended.	Pages 6-12
Ancillary analyses	18	Results of any other analyses performed, including subgroup analyses and adjusted analyses, distinguishing pre-specified from exploratory	n/a
Harms	19	All important harms or unintended effects in each group (for specific guidance, see CONSORT for harms)	n/a
**Discussion**			
Limitations	20	Trial limitations, addressing sources of potential bias, imprecision, and, if relevant, multiplicity of analyses	13,14
Generalisability	21	Generalisability (external validity, applicability) of the trial findings	12,13,18
Interpretation	22	Interpretation consistent with results, balancing benefits and harms, and considering other relevant evidence	12,13
**Other information**			
Registration	23	Registration number and name of trial registry	14
Protocol	24	Where the full trial protocol can be accessed, if available	14
Funding	25	Sources of funding and other support (such as a supply of drugs), the role of funders	18
